# Revisiting the Taxonomy of the Genus *Arcobacter*: Getting Order From the Chaos

**DOI:** 10.3389/fmicb.2018.02077

**Published:** 2018-09-04

**Authors:** Alba Pérez-Cataluña, Nuria Salas-Massó, Ana L. Diéguez, Sabela Balboa, Alberto Lema, Jesús L. Romalde, Maria J. Figueras

**Affiliations:** ^1^Departament de Ciències Mèdiques Bàsiques, Facultat de Medicina, Institut d’Investigació Sanitària Pere Virgili, Universitat Rovira i Virgili, Reus, Spain; ^2^Departamento de Microbiología y Parasitología, CIBUS-Facultad de Biología, Universidade de Santiago de Compostela, Santiago de Compostela, Spain

**Keywords:** *Arcobacter*, *Aliiarcobacter* gen. nov., *Pseudoarcobacter* gen. nov., *Haloarcobacter* gen. nov., *Malacobacter* gen. nov., *Poseidonibacter* gen. nov., taxonomic criteria

## Abstract

Since the description of the genus *Arcobacter* in 1991, a total of 27 species have been described, although some species have shown 16S rRNA similarities below 95%, which is the cut-off that usually separates species that belong to different genera. The objective of the present study was to reassess the taxonomy of the genus *Arcobacter* using information derived from the core genome (286 genes), a Multilocus Sequence Analysis (MLSA) with 13 housekeeping genes, as well as different genomic indexes like Average Nucleotide Identity (ANI), *in silico* DNA–DNA hybridization (*is*DDH), Average Amino-acid Identity (AAI), Percentage of Conserved Proteins (POCPs), and Relative Synonymous Codon Usage (RSCU). The study included a total of 39 strains that represent all the 27 species included in the genus *Arcobacter* together with 13 strains that are potentially new species, and the analysis of 57 genomes. The different phylogenetic analyses showed that the *Arcobacter* species grouped into four clusters. In addition, *A. lekithochrous* and the candidatus species ‘*A. aquaticus*’ appeared, as did *A. nitrofigilis*, the type species of the genus, in separate branches. Furthermore, the genomic indices ANI and *is*DDH not only confirmed that all the species were well-defined, but also the coherence of the clusters. The AAI and POCP values showed intra-cluster ranges above the respective cut-off values of 60% and 50% described for species belonging to the same genus. Phenotypic analysis showed that certain test combinations could allow the differentiation of the four clusters and the three orphan species established by the phylogenetic and genomic analyses. The origin of the strains showed that each of the clusters embraced species recovered from a common or related environment. The results obtained enable the division of the current genus *Arcobacter* in at least seven different genera, for which the names *Arcobacter*, *Aliiarcobacter* gen. nov., *Pseudoarcobacter* gen. nov., *Haloarcobacter* gen. nov., *Malacobacter* gen. nov., *Poseidonibacter* gen. nov., and Candidate ‘*Arcomarinus’* gen. nov. are proposed.

## Introduction

The genus *Arcobacter* was created by Vandamme et al. (1991) to accommodate Gram-negative, curved-shaped bacteria belonging to two species *Campylobacter cryaerophila* (now *Arcobacter cryaerophilus*) and *Campylobacter nitrofigilis* (now *A. nitrofigilis*), considered atypical campylobacters due to their ability to grow at lower temperatures (15°C–30°C) and without microaerophilic conditions (Vandamme et al., 1991). The latter species was selected as the type species for the new genus (Vandamme et al., 1991). One year later the genus was enlarged with the addition of two new species, *A. skirrowii* with an animal origin being isolated from aborted ovine, porcine and bovine fetuses, and from lambs with diarrhea, and *A. butzleri*, which was recovered from cases of human and animal diarrhea (Vandamme et al., 1992). Another two new species were incorporated into the genus in 2005. *A. halophilus* was isolated from water from a hypersaline lagoon in Hawaii (Donachie et al., 2005), and *A. cibarius* was isolated from broiled carcasses in Belgium (Houf et al., 2005). These species were assigned to the genus *Arcobacter* on the basis of the 16S rRNA gene similarity (94% and 95% for *A. nitrofigilis* with *A. halophilus* and *A. cibarius*, respectively). However, these values are equal, or even below, the cut-off of 95% for genus definition (Rosselló-Mora and Amann, 2001; Yarza et al., 2008, 2014; Tindall et al., 2010).

From 2009 onward, new species were being described year-by-year, reaching a total number of 27 in 2017. In some of these descriptions, the similarity of the 16S rRNA gene was the decisive character for taxonomic assignation at genus level, although phylogeny based on housekeeping genes (*rpo*B first and then *gyr*B and *hsp*60) was also included as additional, more discriminatory tools for the species (Collado et al., 2009a, 2011; De Smet et al., 2011). Using this approach, *A. molluscorum*, *A. ellisii*, *A. defluvii*, or *A. bivalviorum* were defined, among others (Collado et al., 2009a, 2011; Figueras et al., 2011a,b; Levican et al., 2012), which showed 16S rRNA similarities ranging from 91.1 to 94.7%, not supporting their common affiliation. On the other hand, the most closely related species, which showed a similarity of 99.1% were *A. ellisii* and *A. defluvii* (Collado et al., 2011), giving evidence for the first time of the poor resolution of the 16S rRNA gene for separating closely related species in the genus *Arcobacter*. However, the phylogenetic analysis based on the concatenated sequences of *gyr*B, *rpo*B, and *cpn*60 genes, together with the DNA–DNA hybridization results, clearly supported the existence of these two differentiated taxa (Figueras et al., 2011a). Also in 2011, *A. trophiarum* was discovered from the intestinal tract of healthy fattening pigs, which interestingly showed the closest similarities (≥97.4%) with the other species also recovered from humans or animals, i.e., *A. cryaerophilus*, *A. thereius*, *A. cibarius*, or *A. skirrowii* (De Smet et al., 2011; Figueras et al., 2014; Van den Abeele et al., 2014).

In 2013, the species *A. cloacae* and *A. suis* were described, using a Multilocus Sequence Analysis (MLSA) approach including five housekeeping genes (Levican et al., 2013) for the first time. Simultaneously, and due to the highest 16S rRNA gene similarity with *A. marinus* (95.5%), the species *A. anaerophilus* was incorporated to the genus (Sasi-Jyothsna et al., 2013). However, this species showed atypical characteristics, including lack of motility and obligate anaerobic metabolism, which led to the original description of the genus *Arcobacter* being emended (Sasi-Jyothsna et al., 2013). The most recently described species from shellfish are *A. lekithochrous*, *A. haliotis*, and *A. canalis* (Diéguez et al., 2017; Tanaka et al., 2017; Pérez-Cataluña et al., 2018a). The first one included several isolates recovered from scallop larvae and from tank seawater of a Norwegian hatchery (Diéguez et al., 2017), the second species came from an abalone of Japan (Tanaka et al., 2017) and the third from oysters submerged in a water channel contaminated with wastewater (Pérez-Cataluña et al., 2018a). However, Diéguez et al. (2018) evidenced that the species *A. haliotis* is a later heterotypic synonym of *A. lekithochrous*. Additionally, the low 16S rRNA gene similarity of *A. lekithochrous* with the known *Arcobacter* species (91.0–94.8%) found in the *A. lekithochrous* description made Diéguez et al. (2017) suggest that certain species might belong to other genera and recommend that a profound revision of the genus might clarify the taxonomy.

On the other hand, adding 2.5% NaCl to the enrichment medium and subculturing on marine agar, Salas-Massó et al. (2016) recognized seven potential new species from water and shellfish (mussels and/or oysters), and recovered new isolates of *A. halophilus* and *A. marinus* of which only the type strains had been known. In addition, during the characterization of the most recently described species *A. canalis* (Pérez-Cataluña et al., 2018a) and when trying to define the seven mentioned new species, we observed that the *Arcobacter* species formed several different clusters distant enough to suspect they might correspond to different genera, in agreement with Diéguez et al. (2017).

There are clear criteria for describing new bacterial species (Tindall et al., 2010; Figueras et al., 2011a,b). However, the description of a genus is usually based on a cut-off of <95% similarity in the 16S rRNA gene sequence, and a G+C (% mol) content differing by more than 10% (Rosselló-Mora and Amann, 2001; Yarza et al., 2008; Tindall et al., 2010; Yarza et al., 2014). Nowadays, genomic data like the Average Nucleotide Identity (ANI) and the *in silico* DNA–DNA hybridization (*is*DDH) are used to define bacterial species, although have not yet been fully explored for delineating genera (Konstantinidis and Tiedje, 2005; Goris et al., 2007; Richter and Rosselló-Móra, 2009; Qin et al., 2014; Chun et al., 2018).

A percentage of Average Amino-acid Identity (AAI) ranging from 60 to 80% between the compared genomes of species or strains and a Percentage of Conserved Proteins (POCPs) above 50% has been proposed if they are to belong to the same genus (Konstantinidis and Tiedje, 2005; Qin et al., 2014). Finally, the Relative Synonymous Codon Usage (RSCU) has also been used by some authors to infer evolutionary and ecological links among bacterial species (Ma et al., 2015; Farooqi et al., 2016).

Very recently, Waite et al. (2017) carried out a comparative genomic analysis of the class *Epsilonproteobacteria.* Using 16S and 23S rRNA, 120 single-copy marker proteins and AAI analysis they proposed its reclassification as the new phylum Epsilonbacteraeota. In that study, Waite et al. (2017) also proposed a reclassification of the genus *Arcobacter* as a new Family *Arcobacteraceae*, within the class *Campylobacteria*, order *Campylobacterales*. One weakness of this study, specifically regarding the genus *Arcobacter*, is that only seven validated species were included in the analysis. The new family therefore comprised only the genus *Arcobacter*. However, these findings also support the need for a clarification of the taxonomy of the current genus *Arcobacter*.

The rise of genome sequencing has dramatically changed the landscape of systematics of prokaryotes, improving different aspects such as the identification of species, the functional characterization for resolving taxonomic groups, and the resolution of the phylogeny of higher taxa (Whitman, 2015). It seems clear that the incorporation of genomics into the taxonomy will boost its credibility providing reproducible, reliable, highly informative means to infer phylogenetic relationships among prokaryotes, and avoiding unreliable methods and subjective difficult-to-replicate data (Chun and Rainey, 2014; Chun et al., 2018).

Within this modern taxonomy context, the objective of the present study was to reassess the taxonomy of the known and newly recognized *Arcobacter* species by using a MLSA of 13 housekeeping genes, the whole genome sequences and the derived genomic analysis. The latter analysis included ANI, *is*DDH, AAI, POCP, and RSCU of all *Arcobacter* type strains. In addition, phylogenies based on 16S and 23S rRNA gene sequences were also performed with comparative purposes. The new taxonomic criteria were stable when including whole genome sequences of a second strain of each species or of unassigned sequences obtained from the public databases.

## Materials and Methods

### Bacterial Strains

All 27 valid species included in the genus *Arcobacter* have been studied. They are represented by 39 strains, and 13 strains that are potentially new species (**Table 1**). Furthermore, 50 genomes of *Arcobacter* strains identified at species level were investigated, 39 of which were obtained in our laboratory (27 from known species and 13 from potentially new species) and the others from the public databases^1^^,^^2^. Five genomes that had been deposited as *Arcobacter* sp. in the databases were also included in the study. If there was more than one strain of a known *Arcobacter* species, two representative genomes for each species were included in the analysis. The only exceptions were: *A. acticola* (Park et al., 2016) and *A. pacificus* (Zhang et al., 2015), whose taxonomic positions were only inferred by the phylogenetic analysis of the 16S rRNA gene sequences published in their species descriptions, together with a MLSA of three housekeeping genes (*atp*A, *gyr*B, and *rpo*B) for *A. pacificus* (Zhang et al., 2015; Park et al., 2016). The strains considered potentially new species, and named hereafter as ‘candidate species,’ had been recognized with an MLSA analysis of five housekeeping genes (*atp*A, *gyr*A, *gyr*B, *hsp*60, and *rpo*B) (data not shown).

**Table 1:** Strains used in this study, source of isolation and accession numbers of the available genomes.

Species	Strain	Source	Acc. No. Genome	Species	Strain	Source	Acc. No. Genome
*A. acticola*	KCTC 52212^T^	Seawater	NA^a^	*A. mytili*	T234	Seawater	PDJW00^b^
*A. anaerophilus*	DSM 24636^T^	Estuarine sediment	PDKO00^b^	*A. nitrofigilis*	DSM7299^T^	Marshland plant	NC014166^c^
	IR-1	Utsira aquifer	NZ_JXXG00^c^	*A. pacificus*	DSM 25018^T^	Seawater	NA^a^
*A. aquimarinus*	CECT 8442^T^	Mediterranean Sea	NXIJ00^b^	*A. skirrowii*	LMG 6621^T^	Diarrheic lamb	NXIC00^b^
*A. bivalviorum*	CECT 7835^T^	Mussels	PDKM00^b^		F28	Wild pig	PDJT00^b^
	F118-4	Mussels	PDKL00^b^	*A. suis*	CECT 7833^T^	Pork meat	NREO00^b^
*A. butzleri*	RM4018^T^	Human (Clinical)	NC_009850^c^	*A. thereius*	LMG 24486^T^	Aborted pig foetus	LLKQ01^c^
	ED1	Microbial fuel cell	NC_017187^c^		DU22	Duck cloaca	LCUJ01^c^
*A. canalis*	F138-33	Oyster PNC^e^	NWVW01^b^	*A. trophiarum*	LMG 25534^T^	Piglet feces	PDKD00^b^
	SH-4D_Col1	Unknown	FUYO00^c^		CECT 7650	Chicken cloacal swab	PDJS00^b^
*A. cibarius*	LMG 21996^T^	Broiler, skin	NZ_JABW00^c^	*A. venerupis*	CECT 7836^T^	Clams	NREP00^b^
*A. cloacae*	CECT 7834^T^	Sewage	NXII00^b^	*Arcobacter* sp.	L	Microbial fuel cell	NC_017192^c^
	F26	Mussels	PDJZ00^b^		AF1028	Human feces	JART01^c^
*A. cryaerophilus*	LMG 24291^T^	Aborted bovine foetus	NXGK00^b^		CAB	Marine	Go0012496^d^
*A. defluvii*	CECT 7697^T^	Sewage	NXIH00^b^		LA11	Marine	BDIR01^c^
*A. ebronensis*	CECT 8441^T^	Mussels	PDKK00^b^		LPB0137	Environmental	CP019070^c^
	CECT 8993	Seawater	PDKJ00^b^	
*A. ellisii*	CECT 7837^T^	Mussels	NXIG00^b^	‘*A. aquaticus*’	W112-28	Freshwater PNC^e^	PDKN00^b^
*A. faecis*	LMG 28519^T^	Human septic tank	NZ_JARS00^c^	‘*A. caeni*’	RW17-10	Recycled wastewater	MUXE00^b^
*A. halophillus*	DSM 18005^T^	Hypersaline lagoon	PDJY00^b^	‘*A. hispanicus*’	FW-54	Wastewater	PDKI00^b^
	F166-45	Oyster PNC^e^	PDJY00^b^	‘*A. lacus*’	RW43-9	Recycled wastewater	MUXF00^b^
*A. lanthieri*	LMG 28516^T^	Pig manure	JARU01^c^	‘*A. mediterraneus*’	F156-34	Mussels Alfacs Bay	NXIE00^b^
	LMG 28517	Dairy cattle manure	JARV01^c^	‘*A. miroungae*’	9Ant^f^	Cloaca elephant seal	PDKH00^b^
*A. lekithochrous*	CECT 8942^T^	Great scallop larvae	NZ_MKCO00^b^	‘*A. neptunis*’	F146-38	Mussels Alfacs Bay	PDKG00^b^
	LMG 28652	Abalon	PZYW00^c^	‘*A. porcinus*’	LMG 24487^T^	Aborted pig foetus	LCUH01^c^
*A. marinus*	CECT 7727^T^	Seawater	NXAO01^b^	‘*A. ponticus*’	F161-33	Cockle Alfacs Bay	PDKF00^b^
	F140-37	Clams Alfacs Bay	NWVX01^b^	‘*A. salis*’	F155-33	Oyster PNC^e^	PDKE00^b^
*A. molluscorum*	CECT 7696^T^	Mussels	NZ_NXFY00^b^	‘*A. viscosus*’	F142-34^g^	Mussels PNC^e^	PDKC00^b^
	F91	Mussels	PDJX00^b^	‘*A. vitoriensis*’	FW59^g^	Wastewater	PDKB00^b^
*A. mytili*	CECT 7386^T^	Mussels	NXID00^b^	*Arcobacter* sp.	F2176	Mussels	PDJV00^b^


**^*a*^Genome not available; ^*b*^Genome sequenced in this study; ^*c*^Genome obtained from NCBI database; ^*d*^Genome obtained from JGI Gold atabase; ^*e*^PNC means PobleNou Channel, which is a freshwater channel heavily (geometric mean of *E. coli* counts 4.1 × 10^4^ c.f.u./100ml) contaminated with wastewater where shellfish were exposed for 72h (Salas-Massó et al., 2016, 2018). ^*f*^This strain was obtained from F.J. García from the Laboratorio Central de Veterinaria de Algete, MAGRAMA, Madrid, Spain; ^*g*^These strains were recovered at the Faculty of Pharmacy, University of the Basque Country (UPV-EHU), Vitoria-Gasteiz, Spain, by R. Alonso, I. Martinez-Malaxetxebarria and A. Fernández-Astorga.**

Culturing for genome sequencing was carried out either on blood agar (DIFCO, Madrid, Spain) or marine agar (Scharlau, Sentmenat, Spain) at 30°C in aerobiosis for 24–72 h, depending on the requirements. DNA was extracted using Easy-DNA^TM^ gDNA Purification kit (Invitrogen, Madrid, Spain) following the manufacturer’s instructions. The integrity of the DNA was evaluated by electrophoresis of 10 μl of the sample in a 1.5% agarose gel. The total amount of DNA was quantified using Qubit^TM^ with the dsDNA Broad Range Assay kit (Invitrogen). Paired-end libraries were constructed with 50 ng of DNA using Nextera DNA Library Preparation Kit (Illumina, Lisbon, Portugal) and sequenced with MiSeq platform (Illumina). Sequencing generated 2 × 300 bp paired-end reads. Clean reads were assembled with SPAdes (Nurk et al., 2013) and the CGE assembler (Larsen et al., 2012) in order to select the better assembly. Before depositing the genomes in the NCBI database, FASTA files were screened for eukaryotic and prokaryotic sequences using BLASTn, and for adaptors with VecScreen standalone software^3^. The five housekeeping genes used in the first MLSA analysis (*atp*A, *gyr*A, *gyr*B, *hsp*60, and *rpo*B) were extracted from each genome and compared with the Sanger sequences of these genes obtained originally for the identification of the strain. The existence of a single and identical copy of these genes confirmed that the genomes were not contaminated and belonged to the correct strain. Finally, contigs were deleted if they had less than 200 bp. The genomes were deposited in the GenBank database and **Table 1** lists the accession numbers.

The 55 genomes were annotated with a local installation of Prokka v1.2 (Seemann, 2014) using an e-value of 1e-06. The annotation was performed with Prokka, with the prediction tools Prodigal v2.6 (Hyatt et al., 2010) and ARAGORN v1.2 (Laslett and Canback, 2004). The prediction tool Barrnap v0.6^4^ included in Prokka v1.2 was used for the annotation of rRNA genes. Coding sequences (CDS) were annotated, combining the Rapid Annotation Subsystems Technology (RAST) (Overbeek et al., 2014) using the *classic RAST* scheme and the Annotation Tools of PATRIC server (Wattam et al., 2017). The characteristics of each genome (i.e., N50, number of contigs, number of CDS, G+C content) were obtained from NCBI annotations.

### Analysis of Housekeeping Genes, Ribosomal Genes, and Core Genome

Thirteen housekeeping genes (*atp*A, *atp*D, *dna*A, *dna*J, *dna*K, *fts*Z, *gyr*A, *hsp*60, *rad*A, *rec*A, *rpo*B, *rpo*D, and *tsf*) were obtained from the genomes using BLASTn search. Sequence similarities of housekeeping genes were determined using the MegAlign program (DNASTAR^®^, Madison, WI, United States). Genes were aligned using ClustalW (Larkin et al., 2007) and phylogenies based on individual genes and on the concatenated sequences was constructed with MEGA version 6.0 (Tamura et al., 2013) using the Neighbor-Joining (NJ) and Maximum-Likelihood (ML) algorithms.

The phylogenetic analysis of the core genome was assessed with the Roary software (Page et al., 2015) using 80% as cut-off for the BLASTp search. The core genome alignment was extracted with the latter software and the phylogeny was inferred using SplitsTree version 4.14.2 as described in Sawabe et al. (2007) using SplitsTree version 4.14.2, with a neighbor net drawing and Jukes-Cantor correction (Bandelt and Dress, 1992; Huson and Bryant, 2005).

Furthermore, the 16S and 23S rRNA genes of each genome were obtained using RNammer (Lagesen et al., 2007). In some cases, 16S rRNA gene sequences were obtained in our laboratories by Sanger sequencing or from the GenBank. The similarity of the 16S rRNA genes was calculated using MegAlign version 7.0.0 (DNASTAR^®^, Madison, WI, United States). Phylogenetic trees were reconstructed with MEGA version 6.0 (Tamura et al., 2013) also using the NJ and ML algorithms. Alignments obtained for both genes were visually analyzed in order to localize signature sequences for strains or groups of strains.

### Genomic Indices

In order to ensure the correct assignation at species level of each analyzed genome, the ANI and the *is*DDH were calculated between all the genomes (Konstantinidis and Tiedje, 2005; Richter and Rosselló-Móra, 2009; Qin et al., 2014). The ANIb was calculated using JSpeciesWS (Richter et al., 2016), the resulting matrix was clustered and visualized using ggplot2 2.2.1 package (Wickham, 2009) and the *is*DDH was calculated with the GGDC software using results obtained with the formula 2 (Meier-Kolthoff et al., 2013). Two other indices (AAI and POCP) described for genus classification (Konstantinidis and Tiedje, 2005; Luo et al., 2014; Qin et al., 2014) were calculated among the genomes that corresponded to the type strains of the accepted species and the reference strains of the candidate species. The AAI was calculated with the Lycoming College Newman Lab AAIr Calculator^5^ using the Sequence-Based Comparison Tools output file from RAST (Overbeek et al., 2014). The POCP was determined as described by Qin et al. (2014) using the following parameters to consider a peptide as a conserved protein: an e-value lower than 1e-5 and an identity percentage higher than 40% from an aligned region higher than 50%.

Finally, the RSCU was computed using the Codon Adaptation Index (CAI) developed by Sharp and Li (1987) through the CAIcal web-server (Puigbò et al., 2008). Statistical differences in the RSCU were assessed by a multinomial regression approach using the R software environment (R Core Team, 2015). The principal component analysis (PCA) was performed by the R software environment (R Core Team, 2015, and visualized using ggplot2 2.2.1 and ggfortify 0.4.4 (Wickham, 2009; Horikoshi and Tang, 2015; Tang et al., 2016) or pca3d 0.10 (Weiner, 2017) packages.

### Phenotypic Analysis and Metabolic Inference

Phenotypic characterization of each described species was obtained from this study, from the original descriptions or from the summary published by On et al. (2017). For the potentially new *Arcobacter* species, the phenotype was characterized following the recommended minimal standards described for new taxa of the family *Campylobacteraceae* (Ursing et al., 1994; On et al., 2017) and with complementary tests used in the description of other *Arcobacter* species (Levican et al., 2013).

Inference of the metabolic routes from the genome sequences was performed with the software package Traitar (Microbial Trait Analyzer) (Weimann et al., 2016), using the protein coding genes files obtained with Prokka v1.2 (Seemann, 2014). Traitar software is based on phenotypic data extracted from the Global Infectious Disease and Epidemiology Online Network (GIDEON) and Bergey’s Systematic Bacteriology. The software uses two prediction models: the phypat classifier, which predicts the presence/absence of proteins found in the phenotype of 234 bacterial species; and the phypat+PGL classifier, which uses the same information as the phypat combined with the information of the acquisition and loss of protein families and phenotypes during evolutive events. A total of 67 traits available within the software, related to oxygen requirement, enzymatic activities, proteolysis, antibiotic resistance, morphology and motility and the use of different carbon sources, were tested and the combined results of the two predictors were analyzed using a heat map.

## Results and Discussion

### Strains and Genomes

All the 27 species currently included in the genus *Arcobacter* and 13 candidate species have been investigated in the present study, which has analyzed 55 genomes, 16 of them from the public databases and 39 sequenced in this study (**Tables 1**, **2**). It was not possible to analyze the genomes from *A. acticola* and *A. pacificus* because we were unable to get the type strains of the species. The contigs obtained and the N50 values complied with the recently proposed minimal standards for the use of genomes in taxonomic studies (Chun et al., 2018). The genome size ranged from 1.81 Mb for *A. skirrowii* F28 to 3.60 Mb for *A. lekithochrous* CECT 8942^T^ (**Table 2**). The G+C content ranged from 26.1% in *A. molluscorum* CECT 7696^T^ to 34.9% in ‘*A. aquaticus*’ W112-28. The G+C values agree with the range from 24.6% (which corresponded to the type strain of *A. anaerophilus*) to 31% indicated for the genus *Arcobacter* in the recent emended description by Sasi-Jyothsna et al. (2013). Interestingly, 26 genomes (47.3%) showed the presence of Clustered Regularly Interspaced Short Palindromic Repeats (CRISPRs) and CRISPR-associated genes, related with the immune response of the bacteria.

**Table 2:** Genome characteristics and annotation results. Source of whole genome sequences as indicated in **Table 1**.

Species	No. Contigs	N50 (Kb)	CDS (Total)	CDS (Coding)	RNA Genes	tRNAs	ncRNAs	CRISPR Arrays	G+C (%)	Size (Mb)
*A. anaerophilus* DSM 24636^T^	40	186	2,938	2,922	45	40	2	1	29.9	2.98
*A. anaerophilus* IR1	7	1,179	3,360	3,024	61	47	2	3	30.2	3.25
*‘A. aquaticus’* W112-28^T^	20	370	2,500	2,487	55	45	3	0	34.9	2.53
*A. aquimarinus* CECT 8442^T^	68	75	2,473	2,463	46	42	2	0	26.6	2.46
*A. bivalviorum* CECT 7835^T^	179	461	2,786	2,728	50	41	3	0	28.2	2.75
*A. bivalviorum* F118-4	26	209	2,652	2,652	47	38	3	0	28.1	2.71
*A. butzleri* RM4018^T^	1	–	2,261	2,256	71	54	2	0	27.0	2.34
*A. butzleri* ED1	1	–	2,151	2,145	71	54	2	0	27.1	2.26
*‘A. caeni* RW17-10^T^	59	123	2,357	2,337	58	51	3	0	27.1	2.42
*A. canalis* CECT8984^T^	50	166	2,733	2,720	53	48	2	1	27.3	2.78
*A. canalis* SH-4D_Col1	69	72	2,716	2,663	63	52	2	1	27.1	2.82
*A. cibarius* LMG 21996^T^	44	119	2,156	2,110	68	46	2	0	27.1	2.20
*A. cloacae* CECT 7834^T^	135	135	2,826	2,795	58	51	2	3	26.8	2.78
*A. cloacae* F26	40	218	2,470	2,459	53	44	2	1	26.9	2.51
*A. cryaerophilus* LMG 24291^T^	91	54	2,092	2,081	49	40	3	0	27.2	2.06
*A. defluvii* CECT 7697^T^	80	166	2,921	2,894	57	49	2	2	26.3	2.94
*A. ebronensis* CECT 8441^T^	103	188	3,089	3,072	47	39	3	1	29.2	3.15
*A. ebronensis* W129-34	126	217	3,206	3,171	46	40	3	2	29.2	3.23
*A. ellisii* CECT 7837^T^	135	177	2,875	2,840	64	52	2	1	26.9	2.80
*A. faecis* LMG 28519^T^	55	127	2,429	2,376	76	53	2	1	27.2	2.50
*A. halophilus* DSM 18005^T^	111	56	2,677	2,660	54	46	3	2	27.4	2.75
*A. halophilus* F166-45	90	56	2,879	2,864	59	51	2	2	27.0	2.96
*‘A. hispanicus’* FW54^T^	76	148	2,228	2,207	46	40	3	1	26.4	2.21
‘*A. lacus’* RW43-9^T^	24	295	2,194	2,182	47	40	2	0	26.8	2.22
*A. lanthieri* LMG 28516^T^	29	466	2,223	2,190	73	52	3	1	26.7	2.29
*A. lanthieri* AF1581	24	353	2,199	2,186	88	57	3	0	26.8	2.26
*A. lekithochrous* CECT 8942^T^	436	343	3,628	3,316	88	75	3	0	28.6	3.61
*A. lekithochrous* LMG 28652	82	343	3,499	3,330	61	55	3	0	28.2	3.50
*A. marinus* CECT 7727^T^	162	54	2,809	2,781	55	50	2	0	27.0	2.87
*A. marinus* F140-37	76	67	2,725	2,652	59	48	2	0	27.0	2.78
‘*A. mediterraneus’* F156-34^T^	29	689	2,769	2,750	47	41	3	1	27.3	2.83
‘*A. miroungae’* 9Ant^T^	35	363	1,868	1,847	46	41	2	1	28.1	1.84
*A. molluscorum* CECT 7696^T^	117	121	2,746	2,736	58	49	3	6	26.1	2.76
*A. molluscorum* F91	240	150	2,951	2,889	71	58	3	2	26.3	2.89
*A. mytili* CECT 7386^T^	126	70	2,950	2,934	58	48	3	1	26.3	2.97
*A. mytili* T234	145	37	2,735	2,723	54	48	3	0	26.4	2.77
‘*A. neptunis’* F146-38^T^	36	267	2,627	2,614	57	45	3	0	27.1	2.65
*A. nitrofigilis* DSM 7299^T^	1	–	3,101	3,086	69	55	2	1	28.4	3.19
‘*A. ponticus’* F161-33	24	597	2,632	2,621	46	36	3	0	28.1	2.74
‘*A. porcinus’* LMG 24487^T^	70	123	2,186	2,112	47	41	2	0	27.0	2.14
‘*A. salis’* F155-33^T^	153	169	2,932	2,904	50	43	3	0	29.0	2.93
*A. skirrowii* LMG 6621^T^	62	306	2,029	2,006	48	42	2	2	27.7	1.97
*A. skirrowii* F28	110	40	1,911	1,897	46	41	2	0	27.8	1.81
*A. suis* CECT 7833^T^	122	142	2,646	2,613	57	52	2	0	27.3	2.62
*A. thereius* LMG 24486^T^	2	1,039	1,896	1,883	57	46	2	3	27.0	1.91
*A. thereius* DU22	19	252	2,006	1,983	47	42	2	1	26.8	2.01
*A. trophiarum* CECT 7650	37	152	1,911	1,894	48	37	3	0	28.0	1.90
*A. trophiarum* LMG 25534^T^	266	86	2,167	2,071	49	41	3	0	29.4	2.00
*A. venerupis* CECT 7836^T^	234	182	3,319	3,267	64	52	2	0	28.0	3.28
‘*A. viscosus’* F142-34^T^	82	65	2,772	2,756	55	48	3	1	26.6	2.79
‘*A. vitoriensis’* FW59^T^	144	179	2,617	2,570	53	46	2	0	27.4	2.58
*Arcobacter* sp. CAB	367	20	3,596	3,392	NA	31	NA	NA	28.2	3.48
*Arcobacter* sp. F2176	99	178	3,212	3,186	67	57	2	0	28.1	3.27
*Arcobacter* sp. LA11	53	229	3,006	2,961	49	43	3	0	27.9	3.10
*Arcobacter* sp. LPB0137	1	–	2,731	2,698	85	64	2	0	27.7	2.87
*Arcobacter* sp. L^a^	1	–	2,847	2,834	73	56	2	1	26.6	2.95
*Arcobacter* sp. AF1028^b^	46	148	2,336	2,285	71	51	2	1	27.2	2.41


*^*a*^Genome sequenced in this study; ^*b*^Genome obtained from NCBI database; ^*c*^Genome obtained from JGI Gold database. Our results show that these strains belong to the species. ^*d*^*A. defluvii* and ^*e*^*A. faecis*.*

### Taxonomic and Phylogenetic Analysis

Similarities in the 16S rRNA gene sequences among type and representative strains of the different *Arcobacter* species (all the 27 species currently included in the genus and the 13 new candidate species) showed a wide range of values (**Supplementary Tables S1**, **S4**). They ranged from 90.8% (observed between *A. anaerophilus* and *A. faecis*) to 99.9% (between *A. butzleri and* ‘*A. lacus*’). The lower range of similarity (90.8%) is due to the fact that those species, as occurred with others, were assigned within the genus based on the premise that 16S rRNA gene similarity was higher with any type strain of *Arcobacter* than with other taxa. However, in some cases being below the 95% cut-off value for genus delimitation (Rosselló-Mora and Amann, 2001; Yarza et al., 2008; Tindall et al., 2010; Figueras et al., 2011a,b). It is interesting to point out that 16S rRNA gene sequence similarities among *A. nitrofigilis*, the type species of the genus, and the other described species ranged from 93.2% (with *A. thereius*) to 95.9% (with *A. venerupis*). Furthermore, *A. nitrofigilis* showed higher similarities than the threshold value of 95% with only seven species (*A. acticola*, ‘*A. caeni*,’ *A. cloacae*, *A. defluvii*, *A. ellisii*, *A. suis*, and *A. venerupis*) out of the 27 accepted species. In any case, from the analysis of the similarities in the 16S rRNA gene sequences among the *Arcobacter* species it is clear that this gene has limited value and that other approaches available in the genomic era of taxonomy are needed for their study.

**FIGURE 1:**
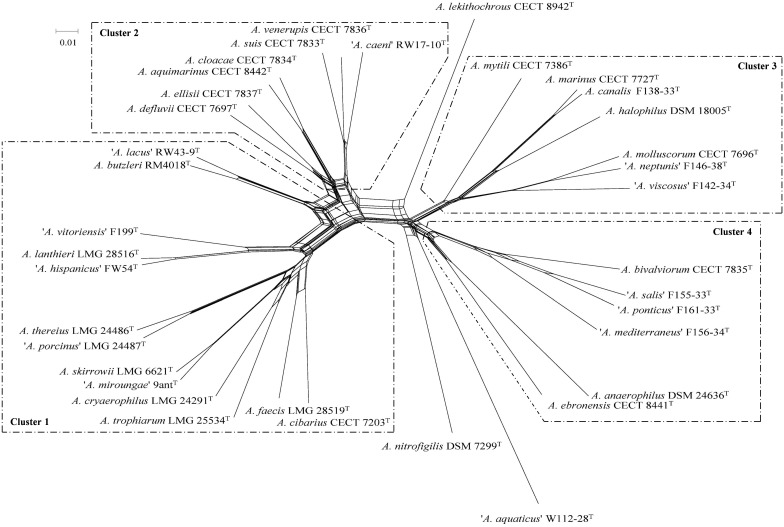
Split decomposition network constructed with the concatenated sequences of 284 core genes from the genomes of 36 type and representative strains of *Arcobacter*. Scale bar, base substitutions per site.

**FIGURE 2:**
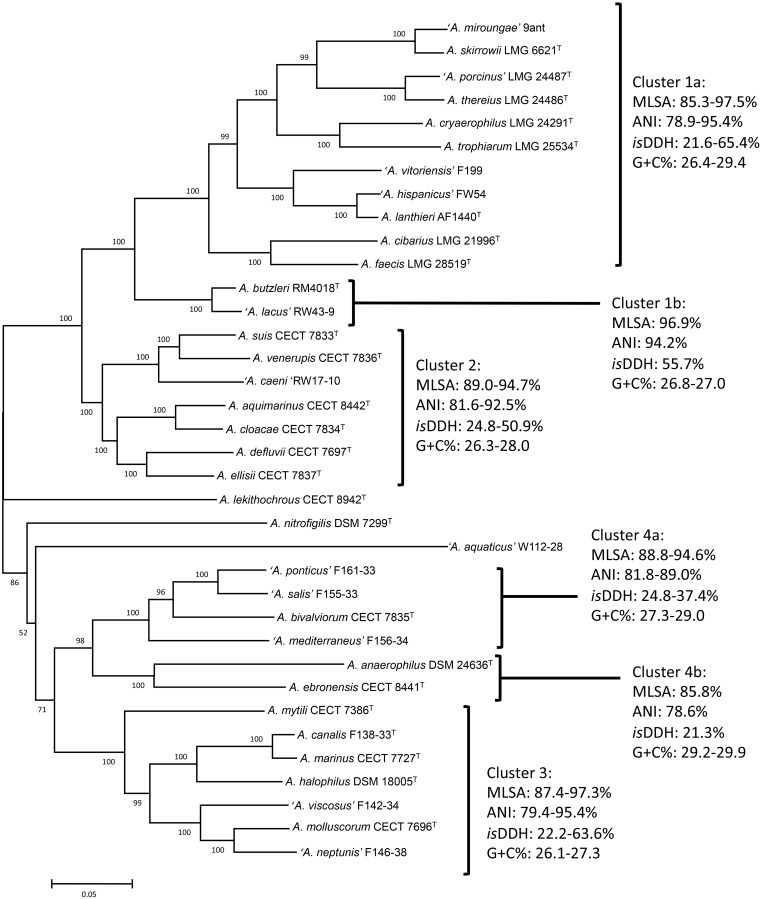
Phylogenetic tree constructed with 36 type and representative strains of *Arcobacter* species based on concatenated sequences of 13 housekeeping genes by the Maximum-Likelihood algorithm (model GTR+G+I). Numbers at nodes denote the level of bootstrap based on 1,000 replicates; only values greater than 50% are shown. Scale bar, base substitutions per site.

Phylogenetic analysis based on the core genome made up of 286 genes (**Figure 1** and **Supplementary Table S5**) and also on the concatenated sequences of 13 housekeeping genes of the representative *Arcobacter* strains (**Figure 2**) revealed that the *Arcobacter* species could be grouped into 4 major monophyletic clusters. Cluster 1, comprised seven validated species: *A. butzleri*, *A. cibarius*, *A. cryaerophilus*, *A. lanthieri*, *A. skirrowii*, *A. thereius*, and *A. trophiarum*, together with *A. faecis* (species described but not validated yet) and five candidate taxa ‘*A. hispanicus*,’ ‘*A. lacus*,’ ‘*A. miroungae*,’ ‘*A. porcinus*,’ and ‘*A. vitoriensis*’ (**Figure 1**). Cluster 2 embraced the species *A. aquimarinus*, *A. cloacae*, *A. defluvii*, *A. ellisii*, *A. suis*, and *A. venerupis*, as well as the non-validated *A. acticola* and the candidatus ‘*A. caeni.*’ Cluster 3 included five species, *A. canalis*, *A. halophilus*, *A. marinus*, *A. molluscorum*, and *A. mytili*, together with two candidates, ‘*A. neptunis*’ and ‘*A. viscosus.*’ Finally, Cluster 4 included the species *A. anaerophilus*, *A. bivalviorum*, and *A. ebronensis*, as well as the candidates ‘*A. mediterraneus*,’ ‘*A. ponticus*,’ and ‘*A. salis.*’ The split decomposition network analysis of the core genome showed that the species *A. lekithochrous* CECT 8942^T^ and *A. nitrofigilis* DSM 7299^T^ appeared as orphan species. Furthermore, with this analysis the candidatus ‘*A. aquaticus’* W112-28 also appeared in a separate branch near to *A. nitrofigilis* DSM 7299^T^. On the other hand, both analyses, MLSA and core genome, confirmed the existence of two sub-clusters in Cluster 1 (again *A. butzleri* and ‘*A. lacus*’ were located in the most distant branch within the cluster), and also two subgroups could be observed in Cluster 4, one comprising the species *A. anaerophilus* and *A. ebronensis*, and the other including the rest of species within this cluster (**Figures 1**, **2**). All the clusters and sub-clusters showed a similarity in the concatenated sequences of the 13 housekeeping genes higher than 85% (**Figure 2**).

Phylogenies based on the 16S and 23S rRNA gene sequences, undertaken with the NJ and ML approacheserealso constructed with comparative purposes. 16S rRNA based tree showed also the four major clusters although less defined (**Supplementary Figure S1A**). Species within Cluster 1, showed 16S rRNA gene sequence similarities ranging from 96.1 to 99.9%. Cluster 2 yielded similarities among species for the 16S rRNA gene between 96.7 and 99.6%, whereas within Cluster 3 ranged between 93.0 and 99.1%. Finally, Cluster 4 included species with a range of 16S rRNA sequence similarity from 94.0 to 99.5%. With the exception of Cluster 3, similarity values within the clusters (>94–95%) were within the classical boundaries for genus assignation in bacterial taxonomy (Rosselló-Mora and Amann, 2001; Yarza et al., 2008, 2014; Tindall et al., 2010; Figueras et al., 2011a,b). Our results agree with those from a recent study by Yarza et al. (2014), who investigated 568 taxa and described a threshold in 16S rRNA sequence identity of 94.5% for genus delineation.

Similar groups and topology, with only minor differences, were obtained when the 23S rRNA gene sequences were used to analyze the phylogeny of the genus (**Supplementary Figure S2**). In this analysis, the recently described species *A. acticola*, and *A. pacificus* could not be included because of the unavailability of the type strains and/or whole genome sequences. The same four major clusters formed in the 23S rRNA gene phylogenetic tree, and the species *A. lekithochrous* and *A. nitrofigilis* appeared also as orphan species (**Supplementary Figure S2**). Within Cluster 1 two subgroups could also be obtained, differentiating the species *A. butzleri* and ‘*A. lacus*’ from the rest of the species. Similarly, the species *A. anaerophilus* and *A. ebronensis* formed a differentiated subgroup in Cluster 4.

The visual analysis of the alignments obtained with the sequences of the 16S and 23S rRNA genes allowed the localization of signature motifs, especially in the 16S rRNA gene, for the different clusters established in the phylogenetic analysis. In these sequences, a total of 16 locations were found, presenting nucleotide combinations characteristic for the clusters (**Supplementary Figure S3**). Some of these motifs were located in helix regions as interactions with proteins of the ribosomal 30S subunit, such as helix 21 (region V4) or helix 28/44 (region V9), and therefore had a considerable level of protection against mutations (Adilakshmi et al., 2008; Kitahara et al., 2012). There are some studies on the presence of signature regions with taxonomic/phylogenetic implications in the ribosomal genes (Martínez-Murcia et al., 1992, 2007; Ue et al., 2011;Řeháková et al., 2014; Martínez-Murcia and Lamy, 2015). Some regions with signature motifs detected in the present study have also shown implications for phylogenetic analysis in cyanobacteria, including regions H15, H17, H21, H22-H23, H41, and H44 (Řeháková et al., 2014). A tree was also constructed weighting such positions (**Supplementary Figure S1B**), which allowed a better definition of the main clusters observed with the whole 16S rRNA sequences although, as expected, differentiation among species within each cluster was lower. Two sub-clusters were observed in Cluster 1, where the species *A. butzleri* and ‘*A. lacus*’ grouped into a well-differentiated branch with respect to the other species in the cluster (**Supplementary Figure S1B**). In this analysis, *A. pacificus* was clearly located in the Cluster 3, whereas in Cluster 4, *A anaerophilus* was the borderline species, while *A. ebronensis* and ‘*A. mediterraneus*’ were located in an independent branch (**Supplementary Figure S1B**). Therefore, the signature motifs described here might be a new tool for identification of the different clusters and/or genus.

### Genomic Indices

The results of the calculations of the ANI and the *is*DDH among the 36 studied genomes are given in the **Supplementary Table S2** and **Supplementary Figure S4**. The results of the ANI and *is*DDH calculations showed that the genomes grouped into the same clusters observed by the analyses of the MLSA of the 13 housekeeping and core genes (**Figures 1**, **2**). Ranges of ANI within each cluster were from 75.2 to 95.4%, whereas *is*DDH values were between 19.5 and 65.4% (**Figure 2** and **Table 3**). These results confirm the phylogenetic analysis for the 13 new candidate species because all of them showed ANI and *is*DDH values of <96% and <70%, respectively, which are the cut-off values proposed for the delineation of new species (Konstantinidis and Tiedje, 2005; Goris et al., 2007; Richter and Rosselló-Móra, 2009; Figueras et al., 2017). As discussed in other studies, the ANI and *is*DDH indices provided reliable information for the delineation of *Arcobacter* species and are also included in the minimal guidelines to define species using genomes (Whiteduck-Léveillée et al., 2015, 2016; Figueras et al., 2017; Chun et al., 2018). Although those indices are not considered useful for delimiting genera, each of the four clusters showed values that ranged between 75.2 and 81.8% as their lowest ANI, which might be the suitable range for separating different, closely related genera. These values are relatively similar to those reported by Qin et al. (2014) that found 68–82% interspecies ANI values among the genera that they studied. Values of ANI obtained for the candidate species ‘*A. aquaticus*’ were lower than the other results, from 70.0% with *A. cryaerophilus* LMG 24291^T^ to 71.9% with *A. bivalviorum* CECT 7835^T^ and more in line with the Qin et al. (2014) results of 68% (**Supplementary Table S2**). In the case of the *is*DDH the lower values among species in the same cluster ranged between 19.5 and 24.8%, and again these might be the levels associated to different genera.

**Table 3:** Intra-cluster similarities (%) obtained for the 16S rRNA gene and for the different genomic indexes analyzed.

	16S RNA gene		MLSA		ANI		*is*DDH		AAI		POCP		G+C% (mol)	
Cluster 1a	96.8–99.8			85.3–97.5	78.9–95.4		21.6–65.4		72.5–95.0		68.2–95.6		26.4–29.4	26.4–29.4
		96.1–99.9	85.3–97.5			77.7–95.4		20.5–65.4		71.5–95.0		67.0–95.6		
Cluster 1b	99.9		96.9		94.2		55.7		93.7		84.3		26.8–27.0	
Cluster 2	96.7–99.6		89.0–94.7		81.6–92.5		24.8–50.9		73.1–93.5		71.7–87.5		26.3–28.0	
Cluster 3	94.2–99.1		87.4–97.3		79.4–95.4		22.2–63.6		67.6–95.7		75.4–91.4		26.1–27.3	
Cluster 4a	96.6–99.5		88.8–94.6		81.8–89.0		24.8–37.4		80.3–83.4		74.4–90.7		27.3–29.0	27.3–29.9
		94.0–99.5		85.8–94.6		75.2–89.0		19.5–37.4		68.7–83.4		71.6–90.7		
Cluster 4b	96.9		85.8		78.6		21.3		78.4		77.9		29.2–29.9	


With the aim of confirming if the clusters observed might represent different genera, as suggested by the phylogenetic analyses, the similarity indices AAI and POCP were also calculated (**Supplementary Table S3**). In agreement with the 60–80% AAI that have been described for species belonging to the same genus (Konstantinidis and Tiedje, 2005) all our clusters showed lower ranges of between 67.6 to 80.3% (**Table 3**). All the clusters also complied with the POCP proposed for genus separation above 50% (Luo et al., 2014; Qin et al., 2014) because as shown in **Table 3** all clusters showed the lowest values from 67.0 to 75.4%.

It is widely known that synonymous codon usage varies among organisms and that it is related to differences in G+C content, replication strand skew, or gene expression (Suzuki et al., 2008; Farooqi et al., 2016). The interaction of these factors may vary among species depending on their evolutionary process (Ma et al., 2015). It has also been suggested that the extent of codon usage bias plays a role in the adaptation of prokaryotic organisms to their environments and lifestyles (Botzman and Margalit, 2011). To analyze the overall codon usage trends of the *Arcobacter* species, the frequencies of the different codons were obtained from the whole genomes and the RSCU was computed using the CAI, which is a useful tool for estimating codon usage bias (Ma et al., 2015; Farooqi et al., 2016). A first finding was that all the *Arcobacter* species presented a preferential use of the codons finishing in A or T (**Supplementary Figure S5**), which might be expected due to their low G+C% content. The characteristic pattern showed by *A. aquaticus* is noteworthy (**Supplementary Figure S5**), which supports its differentiation from the other species in Cluster 3 as well as its unique taxonomy. Such difference was the only statistically significant (*p* < 0.05) in the multinomial regression analysis carried out.

**FIGURE 3:**
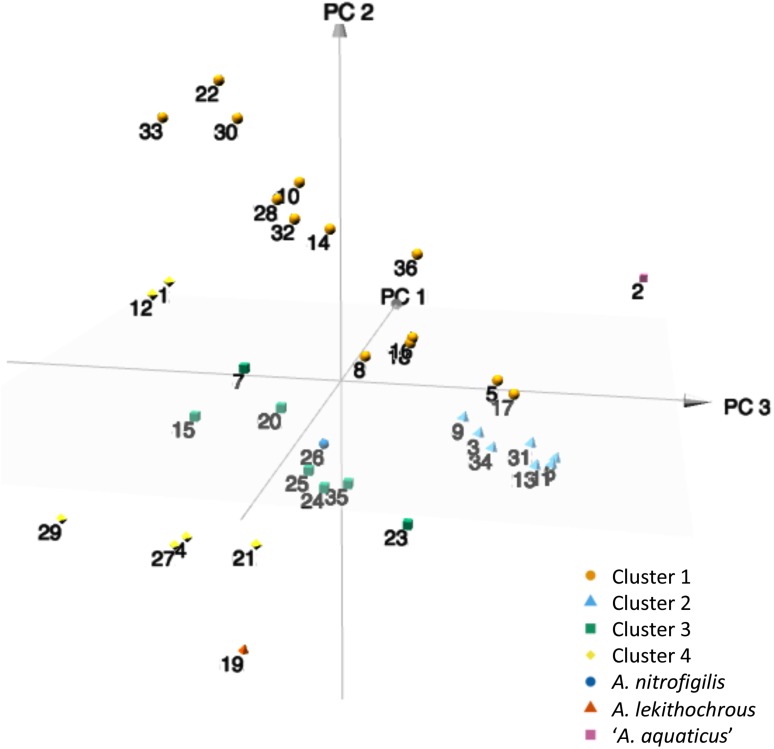
3D plot of the three major axes generated by principal component analysis (PCA) of the RSCU values computed for the 36 type and representative strains of *Arcobacter* species. 1, *A. anaerophilus* DSM 24636^T^; 2, ‘*A. aquaticus*’ W112-28; 3, *A. aquimarinus* CECT 8442^T^; 4, *A. bivalviorum*; 5, *A. butzleri* RM4018^T^; 6, ‘*A. caeni*’ RW17-10; 7, *A. canalis* CECT 8984^T^; 8 *A. cibarius* LMG 21996^T^; 9, *A. cloacae* CECT 7834^T^; 10, *A. cryaerophilus* LMG 24291^T^; 11, *A. defluvii* CECT 7697^T^; 12, *A. ebronensis* F128-2^T^; 13, *A. ellisii* CECT 7837^T^; 14, *A. faecis* AF1078^T^; 15, *A. halophilus* DSM 18005^T^; 16, ‘*A. hispanicus*’ FW54; 17, ‘*A. lacus*’ RW43-9; 18, *A. lanthieri* AF1440^T^; 19, *A. lekithochrous* LFT1.7^T^; 20, *A. marinus* CECT 7727^T^; 21, ‘*A. mediterraneus*’ F156-34; 22, ‘*A. miroungae*’ 9Ant; 23, *A. molluscorum* CECT 7696^T^; 24, *A. mytili* W112-28; 25, ‘*A. neptunis*’ F146-38; 26, *A. nitrofrigilis* DSM 7299^T^; 27, ‘*A. ponticus*’ F161-33; 28, ‘*A. porcinus*’ LMG 24487; 29, ‘*A. salis*’ F155-33; 30, *A. skirrowii* LMG 6621^T^; 31, *A. suis* CECT 7833^T^; 32, *A. thereius* LMG 24486^T^; 33, *A. trophiarum* LMG 25534^T^; 34, *A. venerupis* CECT 7836^T^; 35, ‘*A. viscosus*’ F142-34; 36, ‘*A. vitoriensis*’ F199.

Next, the codon usage trends were analyzed by PCA to reveal possible evolutionary relationships. Interestingly, different groups of strains could be observed in the three-dimensional graphic (**Figure 3**), which correlated with those clusters established in the different phylogenetic analyses, as shown above. As reported previously for different species of *Mycoplasma* (Marenda et al., 2005; Ma et al., 2015), PCA provides an additional pathway to investigate the evolutionary direction of the *Arcobacter* species. In addition, similarities in the synonymous codon usage patterns might reflect similar lifestyles (pathogenic vs. non-pathogenic) and adaptation to certain environments (marine water, shellfish, etc.).

### Metabolic Inference and Phenotypic Analysis

Phylogenetic and genomic analysis confirmed the existence of four clusters among the validated and candidate *Arcobacter* species, which comply with the cut-off values established for the differentiation of independent genera. A thorough phenotypic analysis was therefore carried out to determine if the description of new taxa at genus level was possible or if such clusters were only clades or genomovars within the genus *Arcobacter*. In fact, this is what has occurred in a recent polyphasic study of 52 *A. cryaerophilus* strains (including genome information) in which, despite four different genomospecies being recognized, the phenotypic characterization did not allow their differentiation into separate species and were therefore considered genomovars (Pérez-Cataluña et al., 2018a).

Phenotypic inference using Traitar confirmed the lack of reaction of *Arcobacter* species to most of the tests commonly used for bacterial identification (**Supplementary Figure S6**). Thus, all the type and representative strains rendered negative results, regardless of the predictor employed, for use as the sole carbon source of sugars (D-Mannitol, D-Mannose, Salicin, or Trehalose, among others) and carboxylic acids (Citrate or Malonate). Such results have been previously reported in the original descriptions of the species (see review of On et al., 2017). On the other hand, there was some incongruence between results from Traitar and those obtained by classical characterization for some tests, including growth on MacConkey agar or urea hydrolysis (data not shown). A possible explanation is related with the macro-accuracy of the predictors employed in the Traitar analysis (82.6–85.5%), as reported in the original description of the microbial trait analyzer (Weimann et al., 2016). The fact that some of the *Arcobacter* species studied are halophilic cannot be ignored, since some of the media usually employed in the wet-lab characterization are developed for non-halophilic microorganisms.

The heat maps built from the combined results of both predictors in the Traitar analysis revealed the existence of similarity groups regarding the metabolic characteristics of the *Arcobacter* type strains (**Supplementary Figure S6**). In most case, clustering of strains supported the groups obtained with genomic tools, although some incongruence was also observed, such as for *A. butzleri* (better related here to *A. defluvii, A. ellisii* or *A. cloacae*), *A. mytili* (closest Traitar species ‘*A. caeni*’) or *A. venerupis* (forming a branch with *A. ebronensis* and ‘*A. ponticus*’). In any case, Traitar might be helpful as a first-step method for phenotypic inference, although further verification should be made, especially in environmental bacterial species with special growth requirements (i.e., halophilic conditions).

A deep review of the characteristics reported in the original descriptions of the *Arcobacter* species, together with results obtained in our respective laboratories, allowing phenotypic traits to differentiate the clusters established by the phylogenetic and genomic analyses (**Table 4**). Growth at 37°C in microaerophilic condition, the halophilic character, the ability to grow in presence of glycine, safranin, oxgall, or triphenyltetrazolium chloride (TTC), the presence of some enzymatic activities, such as catalase, urease or indoxyl acetate hydrolysis, and resistance to cefoperazone among others, were the main differentiating traits. Most of these characters are included in the minimal standards for describing new species in the families *Campylobacteraceae* and *Helicobacteraceae* (On et al., 2017), and they should, therefore, also be maintained for the new family *Arcobacteraceae* proposed by Waite et al. (2017), once this taxonomical change is validated. The phenotypic differentiation proposed in **Table 4** enabled to further describe the new genera that corresponded to the different clusters of *Arcobacter* species determined in the present study.

**Table 4:** Differential phenotypic traits among the different clusters of *Arcobacter* species obtained on the basis of the characteristics of the type and representative strains of the species included in each group.

Test	*A. nitrofigilis*	Cluster 1	Cluster 2	Cluster 3	Cluster 4	*A. lekithochrous*	*A. aquaticus*
Growth at/on							
CO_2_ 37°C	–	V	V	+	V	–	+
0.5% NaCl	–	+^a^	+	V	–^b^	–^c^	+
4% NaCl	+	–	–	+	+	–	–
1% Glycine	–	V	–	V	V	–	–
0.05% Safranin	–	+	V	V	V	+	+
0.04 TTC	–	V	–	–	–	+	–
1% Oxgall	–	V	V	–	–^d^	–	–
CCDA	–	V	V	–^e^	–	+	+
Enzymatic activities							
Catalase	–	+^f^	+	V	V	+	–
Urease	+	–	V	–	– ^d^	–	–
Indoxyl acetate hydrolysis	+	+^f^	+	V	V	–	–
Nitrate reduction	+	V	+	–^g^	V	–	–
Resistance to cefoperazone (64 mg/l)	ND	V	–	V	–	–	+


*+, positive result; -, negative result; V, variable result in all the species of the cluster; ^*a*^With the exception of *A. skirrowii*; ^*b*^With the exception of *A. pacificus*; ^*c*^*A. lekithochrous* needs sea salts to grow; ^*d*^With the exception of *A. ebronensis*; ^*e*^With the exception of *A. molluscorum*; ^*f*^With the exception of *A. cibarius*; ^*g*^With the exception of *A. anaerophilus.* ND, not determined.*

### Stability of the Genomic-Based Clustering

In order to test the stability of the new taxonomical scheme proposed, we analyzed the whole genome sequences using second strains from each species or from unassigned sequences obtained from the public databases. That analysis is shown in **Supplementary Figure S7** and included 55 genomes. These new phylogenetic analyses of the core genome also using a Split network showed that the four clusters were maintained, but the two clusters (Clusters 3 and 4) that include species able to grow in media containing 2.5% NaCl appeared in the right place (**Supplementary Figure S7**). The genome of *Arcobacter* sp. LPB0137 obtained from the NCBI database grouped with the species *A. lekithochrous* CECT 8942^T^, while the genomes *Arcobacter* sp. LA11 and CAB grouped together in a separate branch near to Cluster 4. Interestingly, the ANI and *is*DDH values of 91.4% and 45.8% between strain F2176, previously identified as *A. nitrofigilis* (Figueras et al., 2008), and the type strains of this species along with the phylogenetic position (**Supplementary Figure S7**), revealed that this strain belonged to another potentially new species. Furthermore, strains L and AF1028, deposited at the NCBI database as *Arcobacter* sp. were identified as *A. defluvii* and *A. faecis*, respectively, because they clustered with the type strains of those species (**Supplementary Figure S7**). This was also confirmed by the ANI and *is*DDH results being above 96% and 70%, respectively.

Collado and Figueras (2011), in their review about the epidemiology and clinical significance of the genus *Arcobacter*, reported that these bacteria should be considered quite atypical within the class *Epsilonproteobacteria* because of the great diversity of hosts and habitats from which they have been isolated. In order to show if the clusters obtained have a relationship with their ecological habitat, the origin of each strain is also given in **Supplementary Figure S7**. Despite the fact that only two strains from each species were included in the analysis, each of the clusters embraced species that had been recovered from common or related origins. Cluster 1 included by strains isolated from humans and animals, from wastewater and from broiler skin (*A. cibarius* CECT 7203^T^). The fact that some strains isolated from wastewater that was contaminated by humans or animal excreta, gives evidence of the relationship of these sources. This finding agrees with the high abundance of *Arcobacter* in wastewater and in water contaminated with fecal pollution (Collado et al., 2008, 2010). Among the species of Cluster 1, both by metagenomics analysis or direct plating without enrichment (Fisher et al., 2014; Levican et al., 2016), the species *A. cryaerophilus* was the prevalent species in wastewater, while the species *A. butzleri* is normally predominant in studies that investigate water and food samples of animal origin, such as different types of meats using an enrichment step (Collado et al., 2009b; Collado and Figueras, 2011; Hsu and Lee, 2015; and references therein). So far, only the species *A. cryaerophilus*, *A. thereius*, *A. trophiarum, A. cibarius* or *A. skirrowii* have been recovered from humans or animals (De Smet et al., 2011; Figueras et al., 2014; Van den Abeele et al., 2014) and all these species are as commented in the same cluster.

Cluster 2 included strains from different origins but was dominated by species that came from wastewater, shellfish or food products. In this sense, *A. defluvii* CECT 7697^T^ and ‘*A. caeni*’ RW17-10 were isolated from wastewater, while the strain *A. defluvii* L was recovered from a microbial fuel cell. Strains of *A. defluvii* have also been recovered from shellfish in other studies (Levican et al., 2014; Salas-Massó et al., 2016). The strain *A. suis* CECT 7833^T^ was isolated from pork meat, but other isolates have also been obtained from buffalo milk in Italy (Levican et al., 2013; Giacometti et al., 2015). The other five strains in the cluster were isolated from shellfish, wastewater and seawater (**Table 1** and **Supplementary Figure S7**). The other two clusters (Clusters 3 and 4) included strains isolated from seawater shellfish giving evidence of the marine origin of these clusters. The orphan species (*A. nitrofigilis* DSM7299^T^, *A. lekithochrous* CECT 8942^T^, and ‘*A. aquaticus*’ W112-28) also corresponded to strains isolated from marine environments and their phylogenetic position was close to the two marine clusters (3 and 4).

As indicated in the review by Collado and Figueras (2011), there are many uncultured or not-yet-described species of *Arcobacter*, which have been recognized on the basis of nearly full-length 16S rRNA gene sequences, and which probably outnumber those species that were already known at that time. Their hosts and/or habitats are very diverse and include cod larvae, cyanobacterial mats, activated sludge, tidal and marine sediments, estuarine and river water, plankton, coral, tubeworms, snails, etc. (Collado et al., 2011; and references therein). In the near future new species can be expected to emerge that will reinforce the value of the different genera proposed in this study.

## Conclusion

Genomic information obtained through next-generation sequencing leads to great advances in the systematics of prokaryotes (Whitman, 2015), not only to the general understanding of prokaryotic biology but also for the resolution of the phylogeny of taxa higher than species. Single gene phylogeny, including 16S rRNA gene, has often limitations that analysis of complete genome sequences can overcome. The study aims to use this modern taxonomy approach to clarify the relationships of the diverse *Arcobacter* species.

The results obtained in the present study confirmed the opinion of some authors on the need for a clarification of the taxonomy of the genus *Arcobacter*. The phylogenetic analyses derived from the MLSA of 13 genes and of the core genome as well as the existence of signature regions in the 16S rRNA gene have shown, together with the genomic indexes ANI (75.2–81.8%), *is*DDH (19.5–24.8%), AAI (67.6–80.3%), and POCP (67.0–75.4%), to be useful tools for delimiting several genomic and phylogenetic groups within this genus. The intra-genus ranges and cut-off values established here might also be helpful for future taxonomic studies in other bacterial groups.

Such genomic variability, together with the determination of combinations of differentiating phenotypic traits allowed the division of the current genus *Arcobacter* in at least six different genera for which the names *Aliiarcobacter* gen. nov., *Pseudoarcobacter* gen. nov., *Haloarcobacter* gen. nov., *Malacobacter* gen. nov., and *Poseidonibacter* gen. nov. are proposed. In addition, the candidate species ‘*A. aquaticus*’ also constitutes a new genus for which the name Candidate ‘*Arcomarinus’* gen. nov. is proposed, although such proposal should be formulated in parallel to the formal description of the species.

According to Tindall et al. (2010) “*the type strain of a genus is the most important reference organism to which a novel species has to be compared.*” In the case of the genus *Arcobacter*, the type species has rarely been isolated (Collado et al., 2009b; Toh et al., 2011; Levican et al., 2016; Salas-Massó et al., 2016) and in fact, all the analyses show that *A. nitrofigilis* is an orphan species and the only representative of the genus *Arcobacter*, for which an emended description is provided.

The other genera are described here while taking into account the species validated at the time of writing but with the confidence that the formal description of the candidate species would fit in such descriptions. Thus, the genus *Aliiarcobacter* gen. nov. is described comprising seven species *Aliiarcobacter cryaerophilus* comb. nov., *A. butzleri* comb. nov., *A. skirrowii* comb. nov., *A. cibarius* comb. nov., *A. thereius* comb. nov., *A. trophiarum* comb. nov., *A. lanthieri* comb. nov., and *A. faecis* comb. nov. On the other hand, the genus *Pseudoarcobacter* gen. nov. includes the species *Pseudoarcobacter defluvii* comb. nov., *P. ellisii* comb. nov., *P. venerupis* comb. nov., *P. cloacae* comb. nov., *P. suis* comb. nov., *P. aquimarinus* comb. nov., and *P. acticola* comb. nov. Four species, *Malacobacter halophilus* comb. nov., *M. mytili* comb. nov., *M. marinus* comb. nov., *M. molluscorum* comb. nov., and *M. pacificus* comb. nov. are compiled in the new genus *Malacobacter* gen. nov., whereas the genus *Haloarcobacter* gen. nov. comprises three species *Haloarcobacter bivalviorum* comb. nov., *H. anaerophilus* comb. nov., and *H. ebronensis* comb. nov. Finally, the genus *Poseidonibacter* gen. nov. has a unique species *Poseidonibacter lekithochrous* comb. nov.

### Emended Description of the Genus *Arcobacter* Vandamme et al., 1991 emend. Vandamme et al., 1992 and Sasi-Jyothsna et al., 2013

*Arcobacter* (Ar’co.bac.ter. L. n. *arcus*, bow; Gr. n. *bacter*, rod; M. L. masc. n. *Arcobacter*, bow-shaped rod).

Cells are Gram-negative, curved rods 0.2–0.9 μm in diameter and 1–3 μm long. Coccoid bodies are found in old cultures but are not rapidly produced under aerobic conditions. Motile with a rapid corkscrew motion. Each cell possesses a single polar flagellum. Does not swarm. Chemoorganotrophic. Utilizes organic and amino acids as carbon sources, but not carbohydrates. Respiratory metabolism with oxygen as the terminal electron acceptor; anaerobic growth with aspartate and fumarate, but not with nitrate. Nitrate usually reduced to nitrite. Requires NaCl for growth. Grows at temperatures of 10°C–35°C but not at 42°C. Catalase, oxidase, urease, and nitrogenase positive. Phosphatase, sulfatase and indole negative. Does not hydrolyze esculin, casein, DNA, gelatine, hippurate or starch. Fluorescent pigments are not produced. Unable to grow with glycine (1% wt/vol), safranin (0.05% wt/vol), oxgall (1% wt/vol), or 2,3,5-triphenyltetrazolium chloride (0.04%, wt/vol). Positive for the hydrolysis of indoxyl acetate. Poly-β-hydroxybutyrate not produced.

The base composition of the DNA is 28.1–28.4% G+C as determined from the genomes.

The type species is *Arcobacter nitrofigilis.*

### Description of *Aliiarcobacter* gen. nov.

*Aliiarcobacter* (A.li.i.ar.co.bac’ter, L. pronoun *alius* other, another; N.L. masc. n. *Arcobacter* a bacterial generic name; N.L. masc. n. *Aliiarcobacter* the other *Arcobacter*).

Cells are Gram-negative, curved rods 0.2–0.5 μm in diameter and 1–3 μm long. Motile by single polar flagellum. Does not swarm. Chemoorganotrophic. Oxidase and catalase positive. No growth occur at 4% NaCl. Growth occurs at 15°C–42°C. Carbohydrates are not fermented. Nitrate usually reduced to nitrite. Positive for the hydrolysis of indoxyl acetate and negative for urease. Growth does not occur in the presence 2,3,5-triphenyltetrazolium chloride (0.04%, wt/vol) or glycine (1% wt/vol). Some species may grow in the presence of safranin (0.05% wt/vol) or oxgall (1% wt/vol). Fluorescent pigments are not produced. Some species are sensitive to cefoperazone (64 mg/l). Range of DNA G+C content is 26.4–29.4 mol%.

The type species is *Aliiarcobacter cryaerophilus.*

### Description of *Aliiarcobacter cryaerophilus* comb. nov.

Basonym: *Campylobacter cryaerophila* Neill et al., 1985.

Other synonym: *Arcobacter cryaerophilus* Vandamme et al., 1991.

The description is the same given by Neill et al. (1985). The type strain is A169/B^T^ (= NCTC 1185^T^ = ATCC 43158^T^).

### Description of *Aliiarcobacter butzleri* comb. nov.

Basonym: *Campylobacter butzleri* Kiehlbauch et al., 1991.

Other synonym: *Arcobacter butzleri* Vandamme et al., 1992.

The description is the same given by Vandamme et al. (1992). The type strain is LMG 10828^T^ (= CDC D2686^T^ = ATCC 49616^T^).

### Description of *Aliiarcobacter skirrowii* comb. nov.

Basonym: *Arcobacter skirrowii* Vandamme et al., 1992.

The description is the same given by Vandamme et al. (1992). The type strain is Skirrow 449/80^T^ (= LMG 6621^T^ = CCUG 10374^T^).

### Description of *Aliiarcobacter cibarius* comb. nov.

Basonym: *Arcobacter cibarius* Houf et al., 2005.

The description is the same given by Houf et al. (2005). The type strain is LMG 21996^T^ (= CCUG 48482^T^).

### Description of *Aliiarcobacter thereius* comb. nov.

Basonym: *Arcobacter thereius* Houf et al., 2009.

The description is the same given by Houf et al. (2009). The type strain is LMG 24486^T^ (= CCUG 56902^T^).

### Description of *Aliiarcobacter trophiarum* comb. nov.

Basonym: *Arcobacter trophiarum* De Smet et al., 2011.

The description is the same given by De Smet et al. (2011). The type strain is 64^T^ (= LMG 25534^T^ = CCUG 59229^T^).

### Description of *Aliiarcobacter lanthieri* comb. nov.

Basonym: *Arcobacter lanthieri* Whiteduck-Léveillée et al., 2015.

The description is the same given by Whiteduck-Léveillée et al. (2015). The type strain is AF1440^T^ (= LMG 28516^T^ = CCUG 66485^T^).

### Description of *Aliiarcobacter faecis* comb. nov.

Basonym: *Arcobacter faecis* Whiteduck-Léveillée et al., 2016.

The description is the same given by Whiteduck-Léveillée et al. (2016). The type strain is AF1078^T^ (= LMG 28519^T^ = CCUG 66484^T^).

### Description of *Pseudoarcobacter* gen. nov.

*Pseudoarcobacter* (Pseu.do.ar.co.bac’ter, Gr. adj. *pseudes*, false; N.L. masc. n. *Arcobacter* a bacterial generic name; N.L. masc. n. *Pseudoarcobacter*, false *Arcobacter*).

Gram-negative, cells are rod shaped and motile. Cell size 0.2–0.9 μm in diameter and 0.4–2.2 μm long. Some species may present cells up to 10 μm in length. Oxidase and catalase positive. No growth occurs at 4% NaCl. Growth occurs at 15–37°C, but not at 42°C. Carbohydrates are not fermented. Reduce nitrate to nitrite. Positive for the hydrolysis of indoxyl acetate. Some species may hydrolyze urea. Growth does not occur in the presence 2,3,5-triphenyltetrazolium chloride (0.04%, wt/vol) or glycine (1% wt/vol). Some species may grow in the presence of safranin (0.05% wt/vol) or oxgall (1% wt/vol). Sensitive to cefoperazone (64 mg/l). Range of DNA G+C content is 26.3–28.0 mol%.

The type species is *Pseudoarcobacter defluvii.*

### Description of *Pseudoarcobacter defluvii* comb. nov.

Basonym: *Arcobacter defluvii* Collado et al., 2011.

The description is the same given by Collado et al. (2011). The type strain is SW28-11^T^ (= CECT 7697^T^ = LMG 25694^T^).

### Description of *Pseudoarcobacter ellisii* comb. nov.

Basonym: *Arcobacter ellisii* Figueras et al., 2011b.

The description is the same given by Figueras et al. (2011b). The type strain is F79-6^T^ (= CECT 7837^T^ = LMG 26155^T^).

### Description of *Pseudoarcobacter venerupis* comb. nov.

Basonym: *Arcobacter venerupis* Levican et al., 2012.

The description is the same given by Levican et al. (2012). The type strain is F67-11^T^ (= CECT 7836^T^ = LMG 26156^T^).

### Description of *Pseudoarcobacter cloacae* comb. nov.

Basonym: *Arcobacter cloacae* Levican et al., 2013.

The description is the same given by Levican et al. (2013). The type strain is SW28-13^T^ (= CECT 7834^T^ = LMG 26153^T^)

### Description of *Pseudoarcobacter suis* comb. nov.

Basonym: *Arcobacter suis* Levican et al., 2013.

The description is the same given by Levican et al. (2013). The type strain is F41^T^ (= CECT 7833^T^ = LMG 26152^T^).

### Description of *Pseudoarcobacter aquimarinus* comb. nov.

Basonym: *Arcobacter aquimarinus* Levican et al., 2015.

The description is the same given by Levican et al. (2015). The type strain is W63^T^ (= CECT 8442^T^ = LMG 27923^T^).

### Description of *Pseudoarcobacter acticola* comb. nov.

Basonym: *Arcobacter acticola* Park et al., 2016.

The description is the same given by Park et al. (2016). The type strain is AR-13^T^ (= KCTC 52212^T^ = NBRC 112272^T^).

### Description of *Malacobacter* gen. nov.

*Malacobacter* (Ma.la.co.bac’ter; Gr. n. *malaco*, soft, with soft boy, mollusc; Gr. n. *bacter*, rod; N.L. masc. n. *Malacobacter*, bacteria isolated from molluscs).

Gram-negative, cells are rod shaped and motile. Cell size 0.1–0.6 μm wide and 0.5–3.6 μm long. Oxidase positive and catalase variable among species. Halophilic, no growth can be obtained without NaCl and capable to grow up to 4% NaCl. Growth occurs at 15°C–37°C. Does not grow at 37°C in microaerophilic conditions nor at 42°C in anaerobiosis. Carbohydrates are not fermented. Does not reduce nitrate to nitrite. Negative for the hydrolysis of urea. Some species may hydrolyze indoxyl acetate. Growth does not occur in the presence of oxgall (1% wt/vol) or 2,3,5-triphenyltetrazolium chloride (0.04%, wt/vol). Some species may grow in the presence of glycine (1% wt/vol) or safranin (0.05% wt/vol). Sensitive to cefoperazone (64 mg/l) variable among species. Range of DNA G+C content is 26.1–27.3 mol%.

The type species is *Malacobacter halophilus.*

### Description of *Malacobacter halophilus* comb. nov.

Basonym: *Arcobacter halophilus* Donachie et al., 2005.

The description is the same given by Donachie et al. (2005). The type strain is LA31B^T^ (= ATCC BAA-1022^T^ = CIP 108450^T^).

### Description of *Malacobacter mytili* comb. nov.

Basonym: *Arcobacter mytili* Collado et al., 2009a.

The description is the same given by Collado et al. (2009a). The type strain is F2075^T^ (= CECT 7386^T^ = LMG 24559^T^).

### Description of *Malacobacter marinus* comb. nov.

Basonym: *Arcobacter marinus* Kim et al., 2010.

The description is the same given by Kim et al. (2010), with the exception of variable result among strains for the hydrolysis of the indoxyl-acetate under microaerobic conditions (Salas-Massó et al., 2016). The type strain is CL-S1^T^ (= KCCM 90072^T^ = JCM 15502^T^).

### Description of *Malacobacter canalis* comb. nov.

Basonym: *Arcobacter canalis* Pérez-Cataluña et al., 2018b.

The description is the same given by Pérez-Cataluña et al. (2018b). The type strain is F138-33^T^ (= CECT 8984^T^ = LMG 29148^T^).

### Description of *Malacobacter molluscorum* comb. nov.

Basonym: *Arcobacter molluscorum* Figueras et al., 2011a.

The description is the same given by Figueras et al. (2011a). The type strain is F98-3^T^ (= CECT 7696^T^ = LMG 25693^T^).

### Description of *Malacobacter pacificus* comb. nov.

Basonym: *Arcobacter pacificus* Zhang et al., 2015.

The description is the same given by Zhang et al. (2015). The type strain is SW028^T^ (= DSM 25018T = JCM 17857^T^ = LMG 26638^T^).

### Description of *Haloarcobacter* gen. nov.

*Haloarcobacter* (Ha.lo.ar.co.bac’ter, Gr. n. *halo*, salt; N.L. masc. n. *Arcobacter*, a bacterial generic name; N.L. masc. n. *Haloarcobacter, Arcobacter* salt loving).

Gram-negative, cells are rod shaped and motile. Cell size 0.1–0.5 μm in diameter and 0.9–2.5 μm in length. Oxidase positive and catalase variable among species. Halophilic, growth can be obtained within the range of 0.5% (variable among species) and up to 4% NaCl. Growth occurs at 15–42°C. Growth at 37°C in microaerophilic conditions or at 42°C in anaerobiosis variable among species. Carbohydrates are not fermented. Some species may reduce nitrate to nitrite. Negative for the hydrolysis of urea (with the exception of *H. ebronensis*). Some species may hydrolyze indoxyl acetate. Growth does not occur in the presence of oxgall (1% wt/vol) (with the exception of *H. molluscorum*) or 2,3,5-triphenyltetrazolium chloride (0.04%, wt/vol). No growth on CCDA. Some species may grow in the presence of glycine (1% wt/vol) or safranin (0.05% wt/vol). Sensitive to cefoperazone (64 mg/l). Range of DNA G+C content is 27.3–29.9 mol%.

The type species is *Haloarcobacter bivalviorum.*

### Description of *Haloarcobacter bivalviorum* comb. nov.

Basonym: *Arcobacter bivalviorum* Levican et al., 2012.

The description is the same given by Levican et al. (2012). The type strain is F4^T^ (= CECT 7835^T^ = LMG 26154^T^).

### Description of *Haloarcobacter anaerophilus* comb. nov.

Basonym: *Arcobacter anaerophilus* Sasi-Jyothsna et al., 2013.

The description is the same given by Sasi-Jyothsna et al. (2013). The type strain is JC84^T^ (= KCTC 15071^T^ = MTCC 10956^T^ = DSM 24636^T^).

### Description of *Haloarcobacter ebronensis* comb. nov.

Basonym: *Arcobacter ebronensis* Levican et al., 2015.

The description is the same given by Levican et al. (2015). The type strain is F128-2^T^ (= CECT 8441^T^ = LMG 27922^T^).

### Description of *Poseidonibacter* gen. nov.

*Poseidonibacter* (Po.se.i.do.ni.bac’ter, Gr. n. *Poseidon*, God of the sea; Gr. n. *bacter*, rod; N.L. masc. n. *Poseidonibacter* referring to the marine habitat of this bacteria).

Gram-negative, cells are rod shaped and motile. Oxidase and catalase positive. Halophilic, no growth can be obtained without seawater or the addition of combined marine salts to the medium. Growth occurs at 15°C–25°C, but not at 37°C or 42°C. Range of pH for growth is 6–8. Carbohydrates are not fermented. Reduce nitrate to nitrite. Negative for the hydrolysis of indoxyl acetate and urea. Growth occurs in the presence of safranin (0.05% wt/vol), and 2,3,5-triphenyltetrazolium chloride (0.04%, wt/vol), but not in the presence of glycine (1% wt/vol) sensitive to cefoperazone (30 μg). Possess ubiquinone MK-6 as a respiratory quinone. DNA G+C content is 28.7 mol%.

The type species is *Poseidonibacter lekithochrous*.

### Description of *Poseidonibacter lekithochrous* comb. nov.

Basonym: *Arcobacter lekithochrous* Diéguez et al., 2017.

The description is the same given by Diéguez et al. (2017). The type strain is LFT1.7^T^ (= CECT 8942^T^ = DSM 100870^T^).

## Author Contributions

MF and JR designed the work. AP-C, NS-M, and AD performed the phenotypic and phylogenetic experiments. AP-C and SB carried out the genome sequencing and analysis. AP-C, AL, and JR performed the bioinformatic work. JR, MF, AP-C, and AD wrote the paper.

## Conflict of Interest Statement

The authors declare that the research was conducted in the absence of any commercial or financial relationships that could be construed as a potential conflict of interest.

## Footnotes

1 https://www.ncbi.nlm.nih.gov/genome/

2 https://gold.jgi.doe.gov/

3 ftp://ftp.ncbi.nlm.nih.gov/blast/demo/

4 http://www.vicbioinformatics.com/software.barrnap.shtml

5 http://lycofs01.lycoming.edu/~newman/AAI/
