# Corrigendum: Revisiting the Taxonomy of the Genus *Arcobacter*: Getting Order From the Caos

**DOI:** 10.3389/fmicb.2018.03123

**Published:** 2018-12-21

**Authors:** Alba Pérez-Cataluña, Nuria Salas-Massó, Ana L. Diéguez, Sabela Balboa, Alberto Lema, Jesús L. Romalde, Maria José Figueras

**Affiliations:** ^1^Departament de Ciències Mèdiques Bàsiques, Facultat de Medicina, Institut d'Investigació Sanitària Pere Virgili, Universitat Rovira i Virgili, Reus, Spain; ^2^Departamento de Microbiología y Parasitología, CIBUS-Facultad de Biología, Universidade de Santiago de Compostela, Santiago de Compostela, Spain

**Keywords:** *Arcobacter*, *Aliarcobacter* gen. nov., *Pseudoarcobacter* gen. nov., *Haloarcobacter* gen. nov., *Malacobacter* gen. nov., *Poseidonibacter* gen. nov., taxonomic criteria

In the original article, there was an error. The NCTC number for the strain type *Arcobacter cryaerophilus*, was incorrect. A correction has been made to the **Conclusion, Description of *Aliiarcobacter cryaerophilus* comb. nov.:**

The description is the same given by Neill et al. (1985). The type strain is A169/B^T^ (= NCTC 11885^T^ = ATCC 43158^T^).

There were also typographical errors of some Basonym descriptions.

A correction has been made to the Conclusion, Description of *Pseudoarcobacter ellisii* comb. nov., *Malacobacter mytili* comb. nov., *Malacobacter canalis* comb. nov., *Malacobacter molluscorum* comb. nov and Malacobacter pacificus comb. nov.:

Basonym: *Arcobacter ellisii* Figueras et al., 2011b

Basonym: *Arcobacter mytili* Collado et al., 2009

Basonym: *Arcobacter canalis* Pérez-Cataluña et al., 2018

Basonym: *Arcobacter molluscorum* Figueras et al., 2011a

and, Basonym: *Arcobacter pacificus* Zhang et al., 2016.

After indication of the nomenclature editors for validation of new bacterial names, the term *Aliiarcobacter* has been corrected to *Aliarcobacter* throughout the article.

In the original article, the reference for Zhang et al., 2015 was incorrectly written as Zhang, Z., Yu, C., Wang, X., Yu, S., and Zhang, X. H. (2015), It should be Zhang, Z., Yu, C., Wang, X., Yu, S., and Zhang, X. H. (2016).

The authors apologize for these errors and state that they do not change the scientific conclusions of the article in any way. The original article has been updated.
