# Erratum: Corrigendum: Revisiting the Taxonomy of the Genus *Arcobacter*: Getting Order From the Caos

**DOI:** 10.3389/fmicb.2019.00121

**Published:** 2019-01-23

**Authors:** Frontiers Production Office

**Affiliations:** Frontiers Production Office, Frontiers Media SA, Lausanne, Switzerland

**Keywords:** *Arcobacter*, *Aliarcobacter* gen. nov., *Pseudoarcobacter* gen. nov., *Haloarcobacter* gen. nov., *Malacobacter* gen. nov., *Poseidonibacter* gen. nov., taxonomic criteria

To enable validation of the names of some of the newly proposed taxa described in this article, the original article should not have been updated following the publication of the corrigendum. The original version has now been reinstated.

