# Corrigendum (2): Revisiting the Taxonomy of the Genus *Arcobacter*: Getting Order From the Chaos

**DOI:** 10.3389/fmicb.2019.02253

**Published:** 2019-10-01

**Authors:** Alba Pérez-Cataluña, Nuria Salas-Massó, Ana L. Diéguez, Sabela Balboa, Alberto Lema, Jesús L. Romalde, María J. Figueras

**Affiliations:** ^1^Departament de Ciències Mèdiques Bàsiques, Facultat de Medicina, Institut d'Investigació Sanitària Pere Virgili, Universitat Rovira i Virgili, Reus, Spain; ^2^Departamento de Microbiología y Parasitología, CIBUS-Facultad de Biología, Universidade de Santiago de Compostela, Santiago de Compostela, Spain

**Keywords:** *Arcobacter*, *Aliiarcobacter* gen. nov., *Pseudoarcobacter* gen. nov., *Haloarcobacter* gen. nov., *Malacobacter* gen. nov., *Poseidonibacter* gen. nov., taxonomic criteria

The original paper, which contained a description of five new genera with 25 species (all new combinations) contained many errors that prevented the proposed names from being included in a Validation List in the *International Journal of Systematic and Evolutionary Microbiology*. The List Editors could correct part of the problems, so that the generic names *Pseudarcobacter, Malaciobacter*. *Halarcobacter*, and *Poseidonibacter* and the species assigned as “comb. nov.” to these genera could be validly published [*Oren, A. and Garrity, G. M*. *(2019). List of new names and new combinations previously effectively, but not validly, published. Validation List no. 185. Int. J. Syst. Evol. Microbiol*. *69, 5–9*]. The corrections made are explained in footnotes to the list. However, because of the nature of some of the changes required, the List Editors could not make the corrections for the proposed genus “Aliiarcobacter” and the eight proposed new combinations in the Validation List. The corrigendum published [**Revisiting the Taxonomy of the Genus Arcobacter: Getting Order From the Caos (sic)**, *by Pérez-Cataluña, A., Salas-Massó, N., Diéguez, A. L., Balboa, S., Lema, A., Romalde, J. L., et al. (2018). Front. Microbiol. 9:3123. doi: 10.3389/fmicb.2018.03123*] failed to correct the remaining errors and introduced new problems. This necessitated a new Corrigendum in order to effectively publish the names *Aliarcobacter* and eight “comb. nov.” species to be submitted subsequently for List validation in the *International Journal of Systematic and Evolutionary Microbiology*.

**Description of** ***Aliarcobacter*** **gen. nov**.

*Aliarcobacter* (A.li.ar.co.bac'ter. L. pronoun *alius* other, another; N.L. masc. n. *Arcobacter* a bacterial generic name; N.L. masc. n. *Aliarcobacter* the other *Arcobacter*). Cells are Gram-negative, curved rods 0.2–0.5 mm in diameter and 1–3 mm long. Motile by single polar flagellum. Does not swarm. Chemoorganotrophic. Oxidase and catalase positive. No growth occur at 4% NaCl. Growth occurs at 15°C−42°C. Carbohydrates are not fermented. Nitrate usually reduced to nitrite. Positive for the hydrolysis of indoxyl acetate and negative for urease. Growth does not occur in the presence 2,3,5-triphenyltetrazolium chloride (0.04%, wt/vol) or glycine (1% wt/vol). Some species may grow in the presence of safranin (0.05% wt/vol) or oxgall (1% wt/vol). Fluorescent pigments are not produced. Some species are sensitive to cefoperazone (64 mg/l). Range of DNA GC+C content is 26.4–29.4 mol%. The type species is *Aliarcobacter cryaerophilus*.

**Description of** ***Aliarcobacter cryaerophilus*** **comb. nov**.

Basonym: *Campylobacter cryaerophila* Neill et al. 1985.

The description is the same given by Neill et al. (1985). The type strain is A169/B^T^ (= NCTC 11885^T^ = ATCC 43158^T^).

**Description of** ***Aliarcobacter butzleri*** **comb. nov**.

Basonym: *Campylobacter butzleri* Kiehlbauch et al. 1991.

The description is the same given by Vandamme et al. (1992). The type strain is LMG 10828^T^ (= CDC D2686^T^ = ATCC 49616^T^).

**Description of** ***Aliarcobacter skirrowii*** **comb. nov**.

Basonym: *Arcobacter skirrowii* Vandamme et al. 1992.

The description is the same given by Vandamme et al. (1992). The type strain is Skirrow 449/80^T^ (= LMG 6621^T^ = CCUG 10374^T^).

**Description of** ***Aliarcobacter cibarius*** **comb. nov**.

Basonym: *Arcobacter cibarius* Houf et al. 2005.

The description is the same given by Houf et al. (2005). The type strain is LMG 21996^T^ (= CCUG 48482^T^).

**Description of** ***Aliarcobacter thereius*** **comb. nov**.

Basonym: *Arcobacter thereius* Houf et al. 2009.

The description is the same given by Houf et al. (2009). The type strain is LMG 24486^T^ (= CCUG 56902^T^).

**Description of** ***Aliarcobacter trophiarum*** **comb. nov**.

Basonym: *Arcobacter trophiarum* De Smet et al. 2011.

The description is the same given by De Smet et al. (2011). The type strain is 64^T^ (= LMG 25534^T^ = CCUG 59229^T^).

**Description of** ***Aliarcobacter lanthieri*** **comb. nov**.

Basonym: *Arcobacter lanthieri* Whiteduck-Léveillée et al. 2015.

The description is the same given by Whiteduck-Léveillée et al. (2015). The type strain is AF1440^T^ (= LMG 28516^T^ = CCUG 66485^T^).

**Description of** ***Aliarcobacter faecis* comb. nov**.

Basonym: *Arcobacter faecis* Whiteduck-Léveillée et al. 2016.

The description is the same given by Whiteduck-Léveillée et al. (2016). The type strain is AF1078^T^ (= LMG 28519^T^ = CCUG 66484^T^).

The first Corrigendum stated that the original article had been updated. This action was reversed with the publication of the Erratum. The authors apologize for this error and state that this does not change the scientific conclusions of the article in any way.
