# Supplementary material for: Revisiting the Taxonomy of the Genus Arcobacter: Getting Order From the Chaos
Source: Front Microbiol. 2018 Sep 4;9:2077. doi: 10.3389/fmicb.2018.02077 (PMC6131481; doi:10.3389/fmicb.2018.02077)

Revisiting the taxonomy of the genus *Arcobacter*: getting order from the chaos.

Alba Pérez-Cataluña[1], Nuria Salas-Massó[1], Ana L. Diéguez[2], Sabela Balboa[2], Alberto Lema[2], Jesús L. Romalde[2]* and María José Figueras[1]*.

[1]Departament de Ciéncies Médiques Bàsiques, Facultat de Medicina, IISPV, Universitat Rovira i Virgili, Sant Llorenç 21, 43201 Reus, Spain.

[2]Departamento de Microbiología y Parasitología. CIBUS-Facultad de Biología. Universidade de Santiago de Compostela. 15782, Santiago de Compostela, Spain.

SUPPLEMENTARY MATERIAL

Supplementary Figures S1-S7

Supplementary Figure S1A.- Phylogenetic tree constructed with the near complete sequences (1450 nt) of the 16S rRNA gene of 36 type and representative strains of *Arcobacter* species by the Maximum-Likelihood algorithm (model GTR+G+I). Bootstrap values (expressed as percentages of 1000 replications) greater than 50% are shown at the nodes. Scale bars indicate the number of substitutions per nucleotide position. Bold circles indicate that corresponding nodes were coincident in the tree generated with Neigbour-Joining algorithm. Brackets indicate the similarity range for 16S rRNA gene sequences. The cluster names in the tree are based in the phylogenetic results obtained from MLSA and core genome analyses. During the preparation of this article *A. haliotis* was confirmed as a later heterotypic synonym of *A. lekithochrous*.

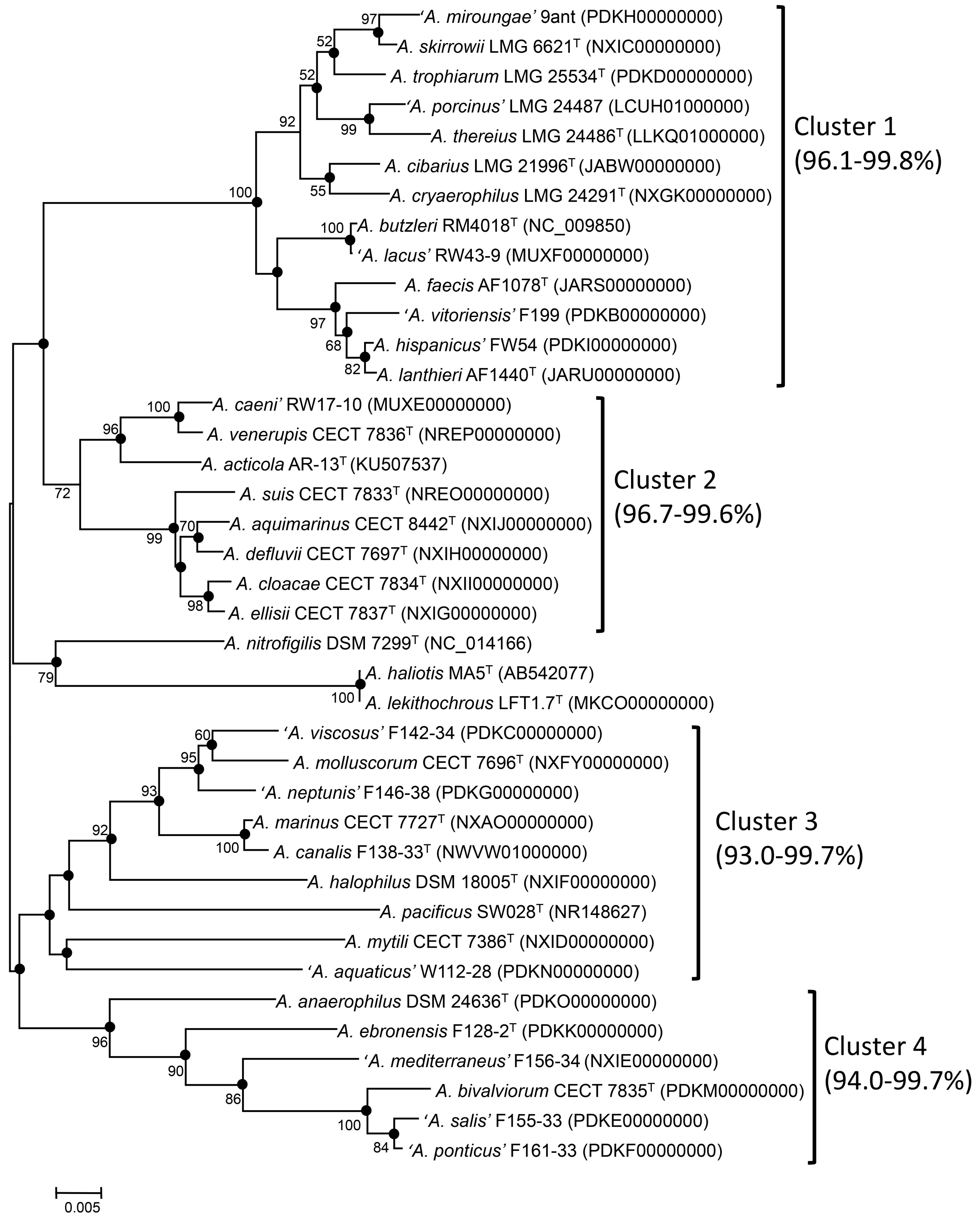

Supplementary Figure S1B.- Tree constructed with the concatenated signature motifs of the 16S rRNA gene for the different clusters. Bootstrap values (expressed as percentages of 1000 replications) greater than 50% are shown at the nodes. Scale bars indicate the number of substitutions per nucleotide position. The cluster names in the tree are based in the phylogenetic results obtained from MLSA and core genome analyses. During the preparation of this article *A. haliotis* was confirmed as a later heterotypic synonym of *A. lekithochrous*.

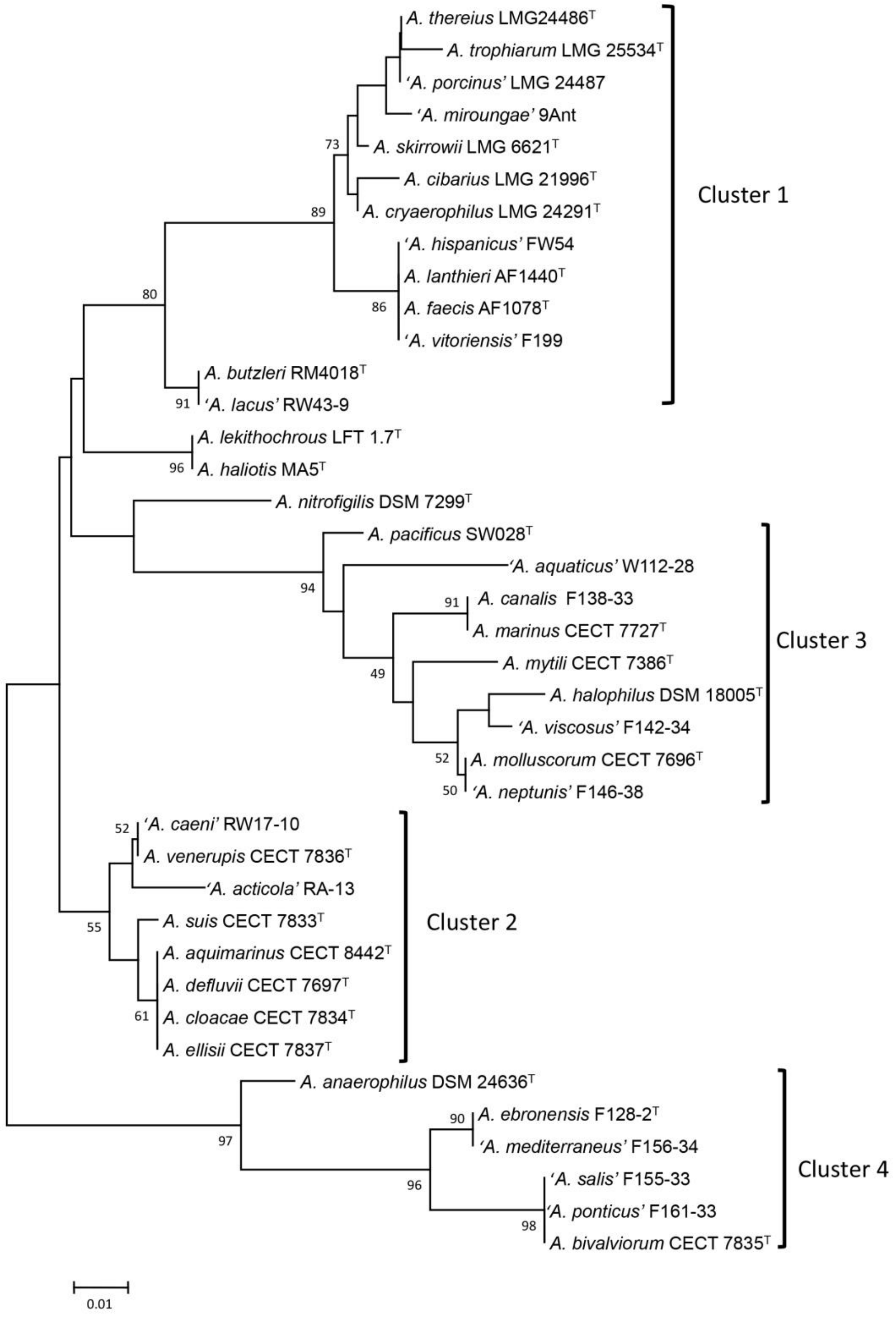

Supplementary Figure S2.- Neighbour joining phylogenetic tree constructed with the 23S rRNA gene sequences (2948 bp) of the type and representative strains of 36 species of *Arcobacter*. Numbers at the nodes indicated bootstrap values >50% obtained by repeating the analysis 1000 times. Scale bar indicates the number of substitutions per nucleotide position. The cluster names in the tree are based in the phylogenetic results obtained from MLSA and core genome analyses.

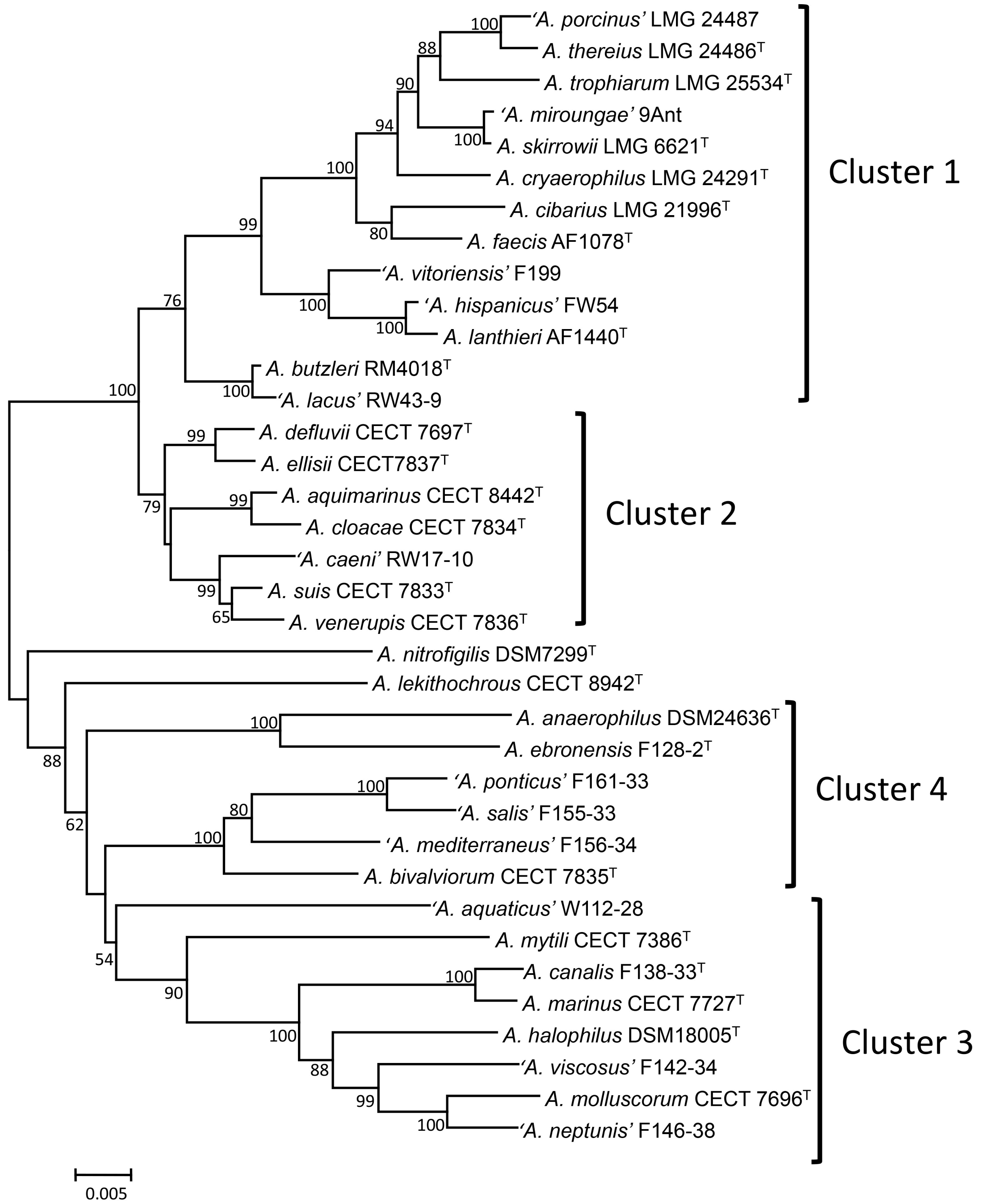

Supplementary Figure S3.- Group specific 16S rRNA gene signatures differentiating the type species of the genus, *Arcobacter nitrofigilis* DSM 7299T and other species of the genus. The numbers at the top give the position in the gene. The location of the signature motifs in the different variable regions and helix of the 16S rRNA gene secondary structure are also indicated. *Escherichia coli* sequence was used as reference to enumerate the nucleotide postitions (Adilakshmi et al., 2008).

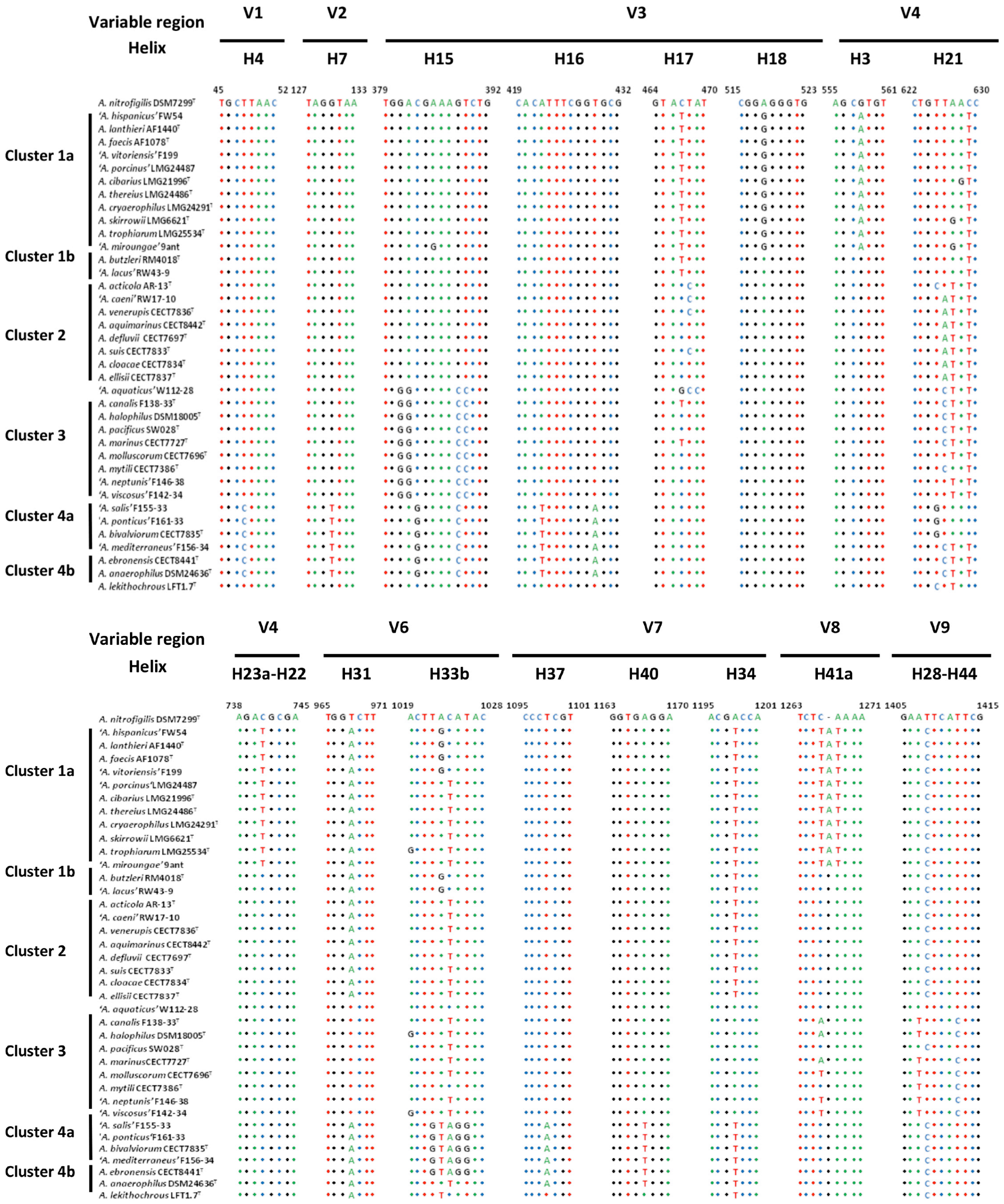

Supplementary Figure S4.- Heatmap representing the similarities (%) among the *Arcobacter* species obtained for ANI (left-down) and isDDH (up-right) indexes.

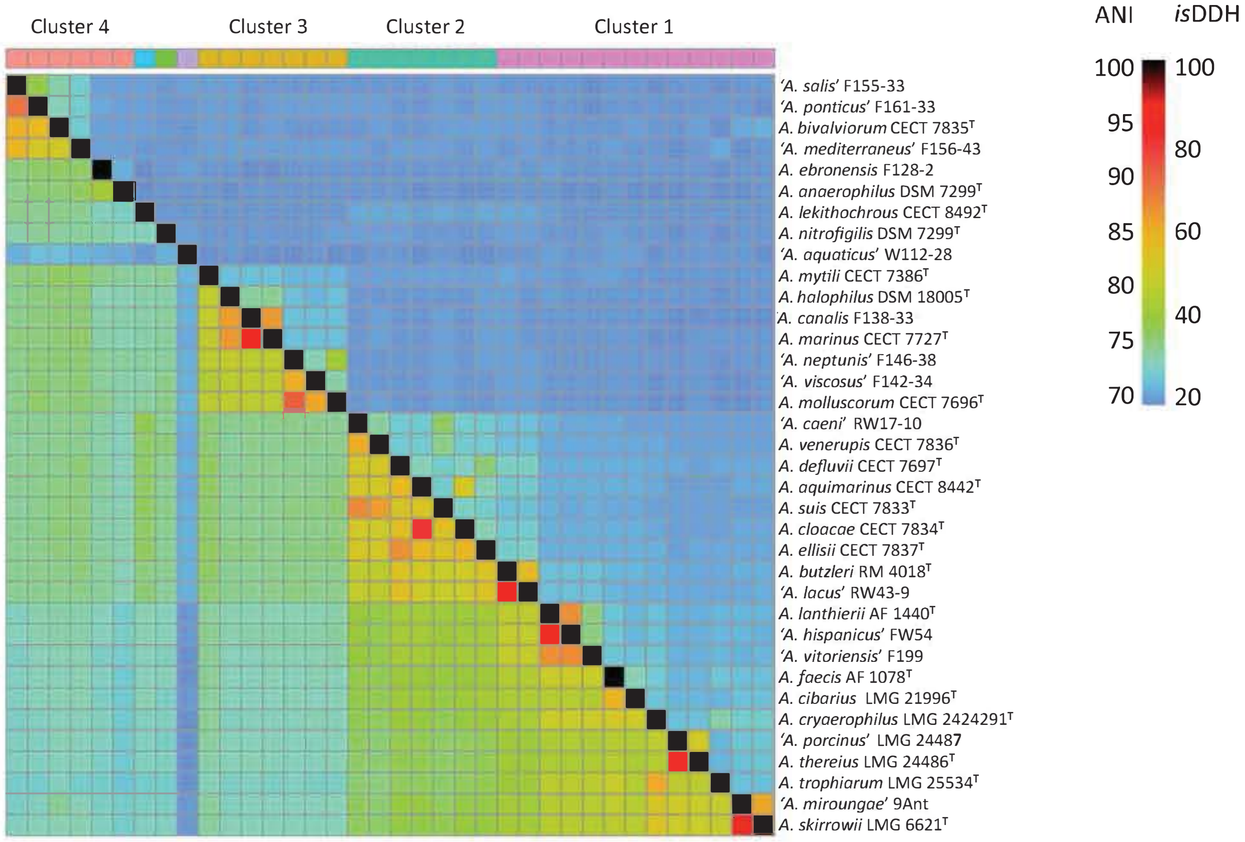

Supplementary Figure S5.- Comparison of the RSCU data of the 59 synonymous codon among the different clusters and orphan species of *Arcobacter*. Lines: blue, cluster 1; red, cluster 2; green, cluster 3; yellow, cluster 4; purple, *A. lekithochrous*; pink, *A. nitrofigilis*; dark blue, *A. aquaticus*.

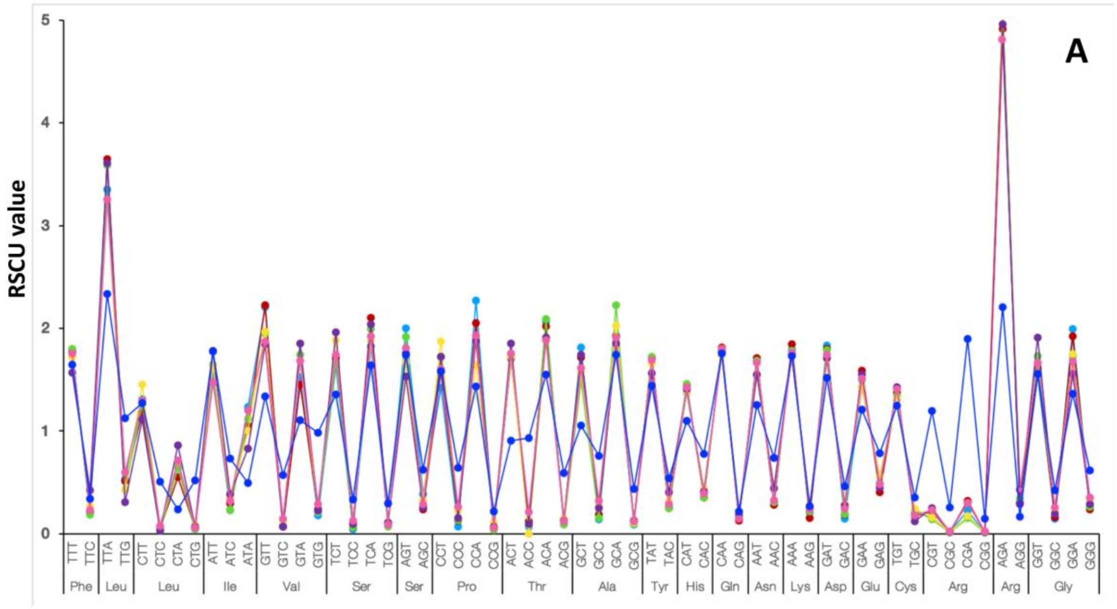

Supplementary Figure S6.- Heatmap obtained with the phenotypic predictor tool Traitar for the type and representative strains of *Arcobacter* species. The origin of the phenotypes prediction (Traitar phypat and/or phypat+PGL classifier) determines the color of the heatmap entries.

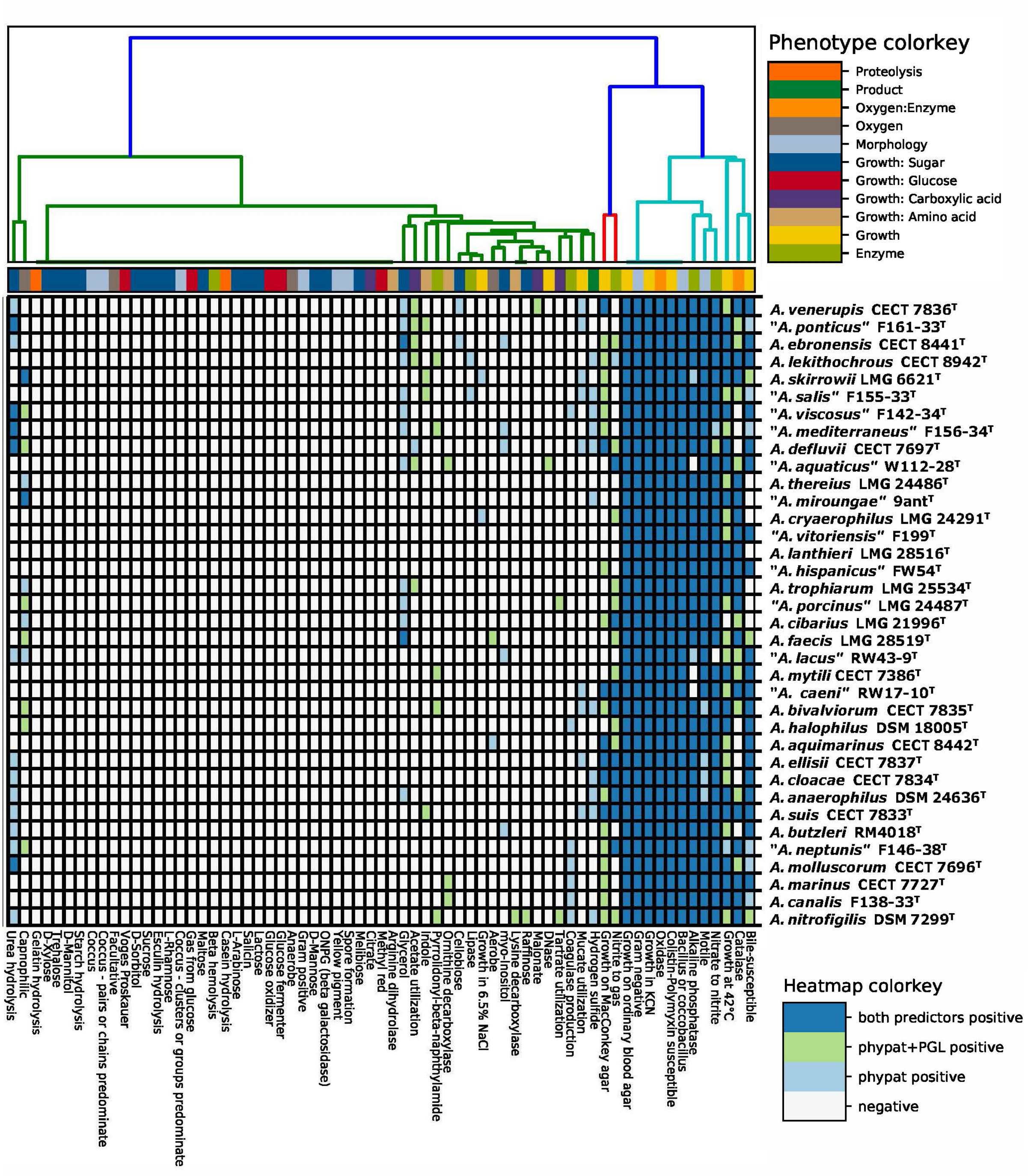

Supplementary figure S7.- Phylogenetic network of the 57 analyzed genomes based on the concatenated sequences of core genes. Scale bar, base substitutions per site.

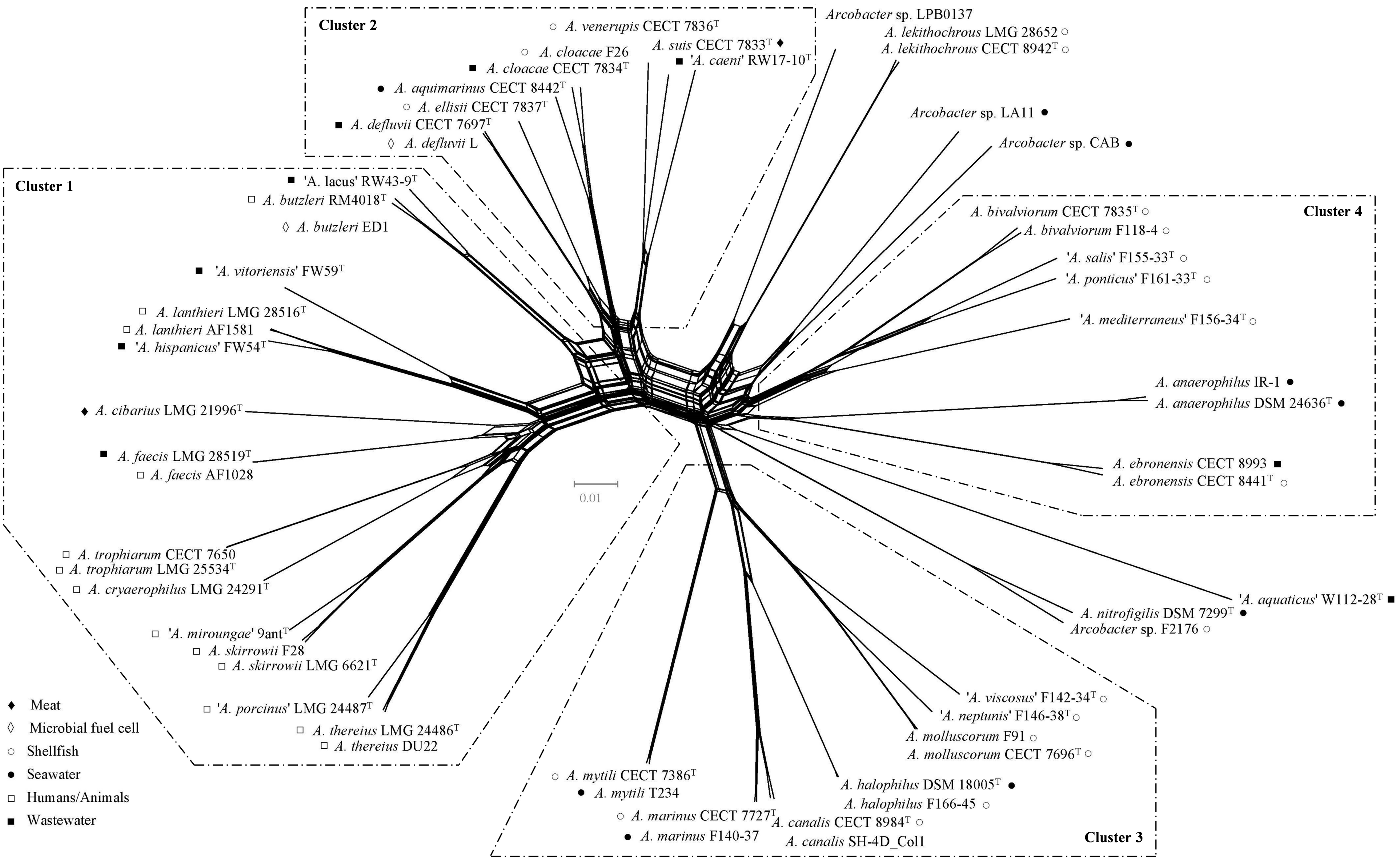

Supplement: Supplementary file 1 [file Presentation_1.PDF]
